# Physico-chemical and key metal data for surface waters and sediments of the Sydney and Hawkesbury estuaries, Australia

**DOI:** 10.1016/j.dib.2019.104255

**Published:** 2019-07-19

**Authors:** Scott J. Markich, Ross A. Jeffree

**Affiliations:** a Aquatic Solutions International, “Point Break”, North Narrabeen Beach, NSW 2101, Australia; b Department of Environmental Sciences, Macquarie University, 12 Wally's Walk, North Ryde, NSW 2109, Australia; c Jeffree Conservation and Research, 45 Casuarina Rd, Alfords Point, NSW 2234, Australia

**Keywords:** Enrichment factor, Ecosystem protection, Dissolved/colloidal, Water quality, Partition coefficient

## Abstract

This article contains general physico-chemical data (salinity, pH, redox potential, temperature, dissolved oxygen, suspended particulate matter (SPM), dissolved organic carbon and chlorophyll *a* concentrations) for surface waters at 15 near-pristine sites in the Hawkesbury Estuary and 24 sites (encompassing a wide range of metal contamination) in the highly urbanized Sydney Estuary, south-eastern Australia. Data on concentrations of five key metals (cadmium (Cd), chromium (Cr), copper (Cu), lead (Pb) and zinc (Zn)) in filtered (<0.2 μm) surface water, suspended particulate matter (>0.2 μm) and surface sediments (<2 mm) at each study site are also provided. The concentrations of Cd, Cr, Cu, Pb and Zn in SPM and sediment at each site were normalised for aluminium (Al) concentration (e.g. Cd/Al), to account for natural variation in particle size and mineralogy. Enrichment factors (EFs) were calculated from these data by dividing the mean metal concentration at each site in the Sydney Estuary, for each environmental matrix (i.e., filtered water, SPM and sediment), by its mean baseline metal concentration from near-pristine reference sites in the adjacent Hawkesbury Estuary. A thorough knowledge of the general physico-chemistry and key metal concentrations in surface waters and sediments in the Sydney Estuary provide a baseline to assess anthropogenic change and better manage estuarine/marine ecosystems.

**Table:** Specifications table

Subject area	*Environmental Science*
More specific subject area	*Estuarine surface water and sediment chemistry*
Type of data	*Tables, Figures*
How data was acquired	*Laser diffraction (Malvern Mastersizer 2000), Water quality sonde (Yellow Springs Instruments 6000UPG), UV–Vis spectrophotometry (Shimadzu UV-2550), Inductively coupled plasma mass spectrometry (HP Agilent 4500), inductively coupled plasma atomic emission spectrometry (Varian Vista AX), Ultraviolet-persulfate oxidation (Tekmar Dohrmann Phoenix 8000 Analyzer), Dry combustion (LECO CNS-2000 Analyzer)*
Data format	*Raw and Analyzed*
Experimental factors	*Sediments were wet sieved through* 2 mm *nylon mesh, oven dried, homogenized in an agate mill and treated with hydrogen peroxide, sodium pyrophosphate and vaporous hydrochloric acid. Suspended particulate matter and sediment were microwave digested with hydrofluoric acid, nitric acid and hydrogen peroxide. Surface water was vacuum filtered (0.*2 μm*) with polycarbonate or polysulfone membrane filters and metals pre-concentrated using an ammonium pyrroliidine dithiocarbamate solvent extraction with mercury back-extraction. Chromium (VI) in filtered surface water was complexed with diphenylcarbazide and pre-concentrated with an isoamyl alcohol solvent extraction. Chlorophyll a was extracted with N,N-dimethylformamide and centrifuged.*
Experimental features	*General physico-chemical and key metal (cadmium, copper, chromium, lead and zinc) analyses of surface waters (filtered + suspended particulate matter) and sediments in the Sydney (highly urbanized) and Hawkesbury (near-pristine) estuaries in south-eastern Australia*
Data source location	*Sydney and Hawkesbury Estuaries, Greater metropolitan Sydney, Australia (33°30*–*53′ S to 151°01*–*18′ E)*
Data accessibility	*Data are available with this article*
Related research article	*Markich, S.J., Jeffree, R.A. 2019. The euryhaline pygmy mussel, Xenostrobus securis, is a useful biomonitor of key metal contamination in the highly urbanized Sydney Estuary, Australia. Environ. Pollut. 52 (2019) 813*–*824.*

**Table:** 

**Value of the data** • Clean sampling techniques have been used for the first time to systematically determine cadmium, chromium, copper, lead and zinc concentrations in the surface waters of the Sydney and Hawkesbury estuaries. • The data permit cadmium, chromium, copper, lead and zinc concentrations in surface water and sediment at sites in the Sydney Estuary to be assessed against national guidelines for the protection of marine ecosystems. • The data provide a quality-assured baseline of key metal contamination in the Sydney Estuary that may be used to discern any changes in metal contaminant status and assist in better managing and protecting marine ecosystems.

## 1 Data

**Fig. 1:**
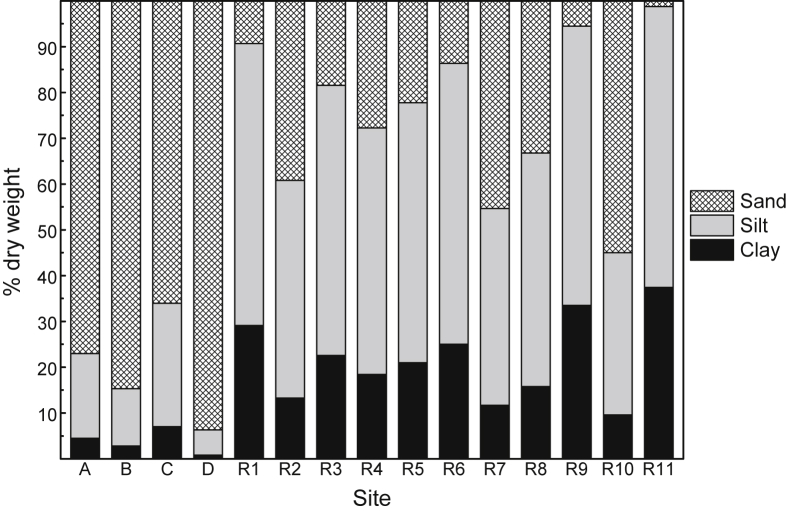
Mean percentage distribution of sand, silt and clay (as dry weight) in surface sediment from study sites in the Hawkesbury Estuary. R1–11 are sites where the mussel, *Xenostrobus securis*, was present, and A, B, C and D are sites where mussels were absent (see Markich and Jeffree [1]) – see Fig. 7 for site locations.

**Fig. 2:**
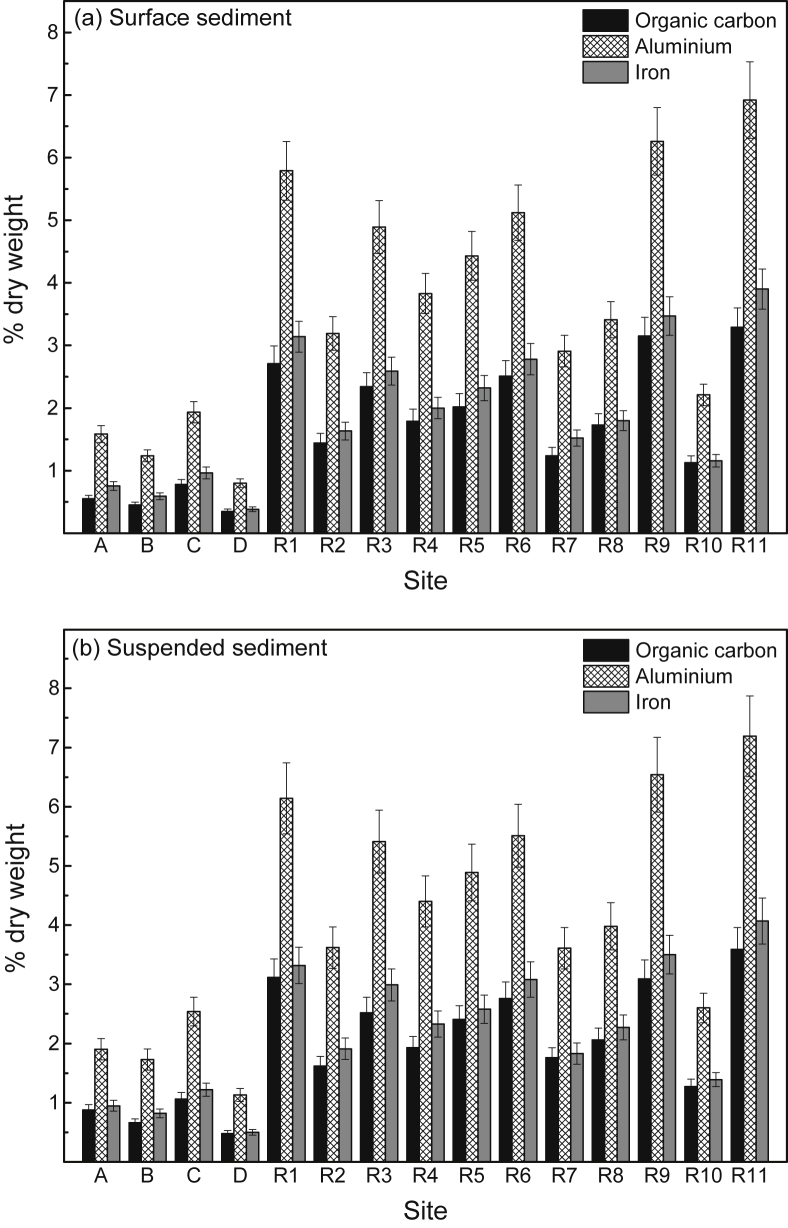
Mean concentrations (% dry weight) (and 84% confidence limits; *p* ≤ 0.05) of particulate organic carbon, aluminium and iron in (a) surface sediment and (b) suspended particulate matter from study sites in the Hawkesbury Estuary. R1–11 are sites where the mussel, *Xenostrobus securis*, was present, and A, B, C and D are sites where mussels were absent (see Markich and Jeffree [1]) – see Fig. 7 for site locations.

**Fig. 3:**
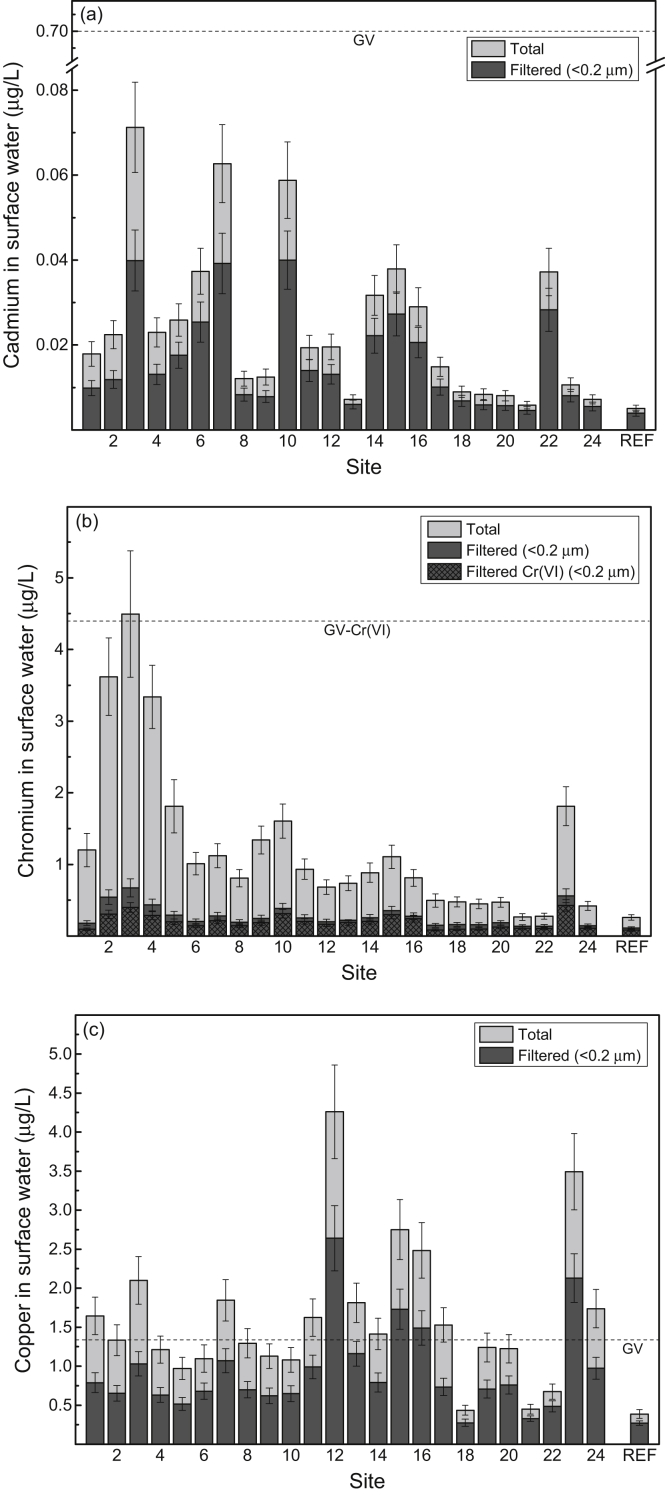

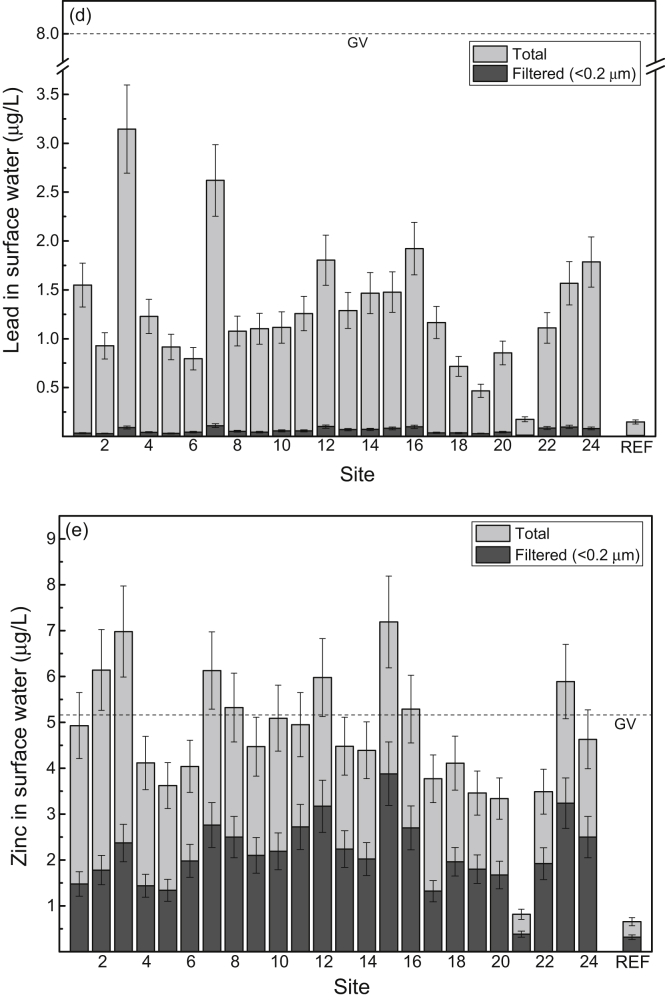
Mean concentrations (μg/L) (and 84% confidence intervals; *p ≤* 0.05) of (a) cadmium, (b) chromium, (c) copper, (d) lead and (e) zinc in surface water from study sites (1–24) in the Sydney Estuary. The mean metal concentrations for the 15 reference sites (REF) in the Hawkesbury Estuary are shown for comparison. Also shown (dashed horizontal lines) are the metal guideline values (GV) for protecting (95% level) marine biota in Australia [6], [13]). See Fig. 7 for site locations and Table 4 (Hawkesbury Estuary) and 5 (Sydney Estuary) for global positioning system (GPS) coordinates.

**Fig. 4:**
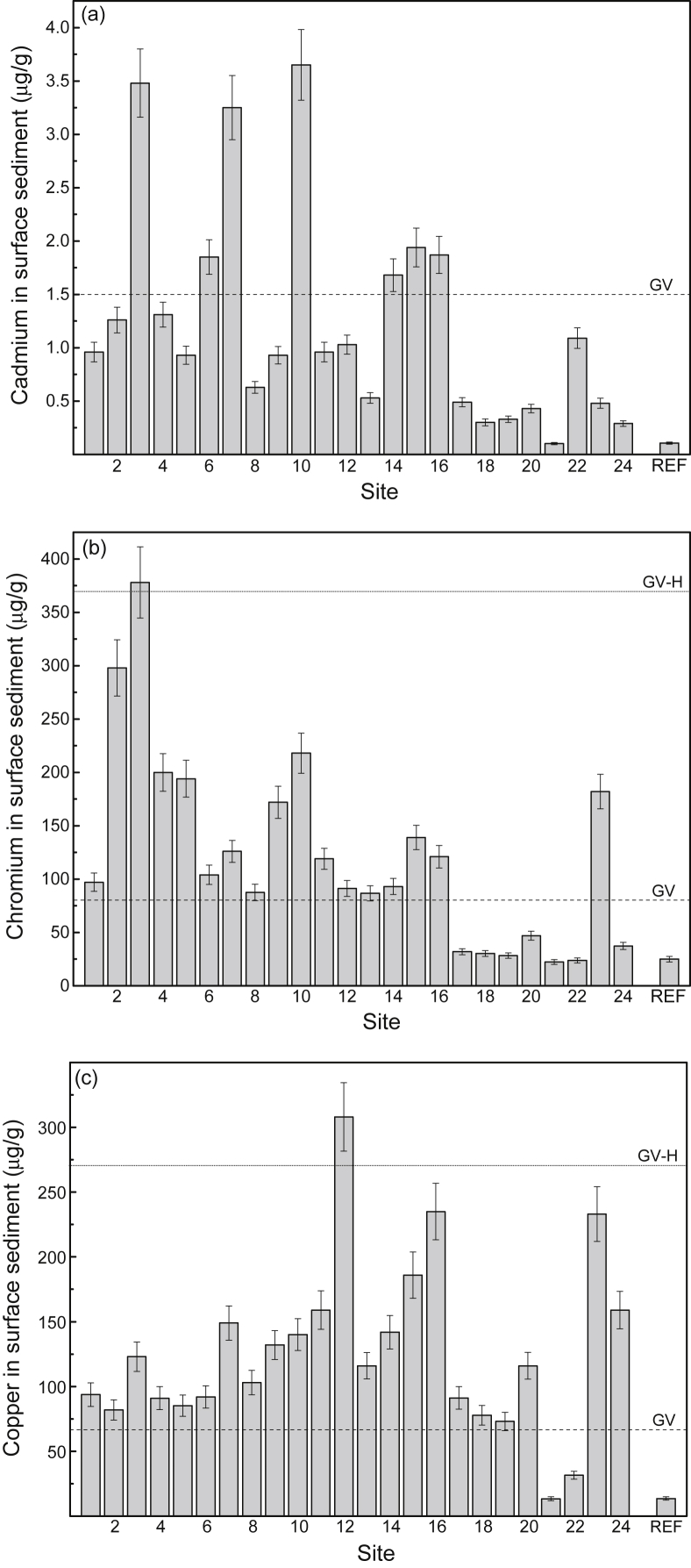

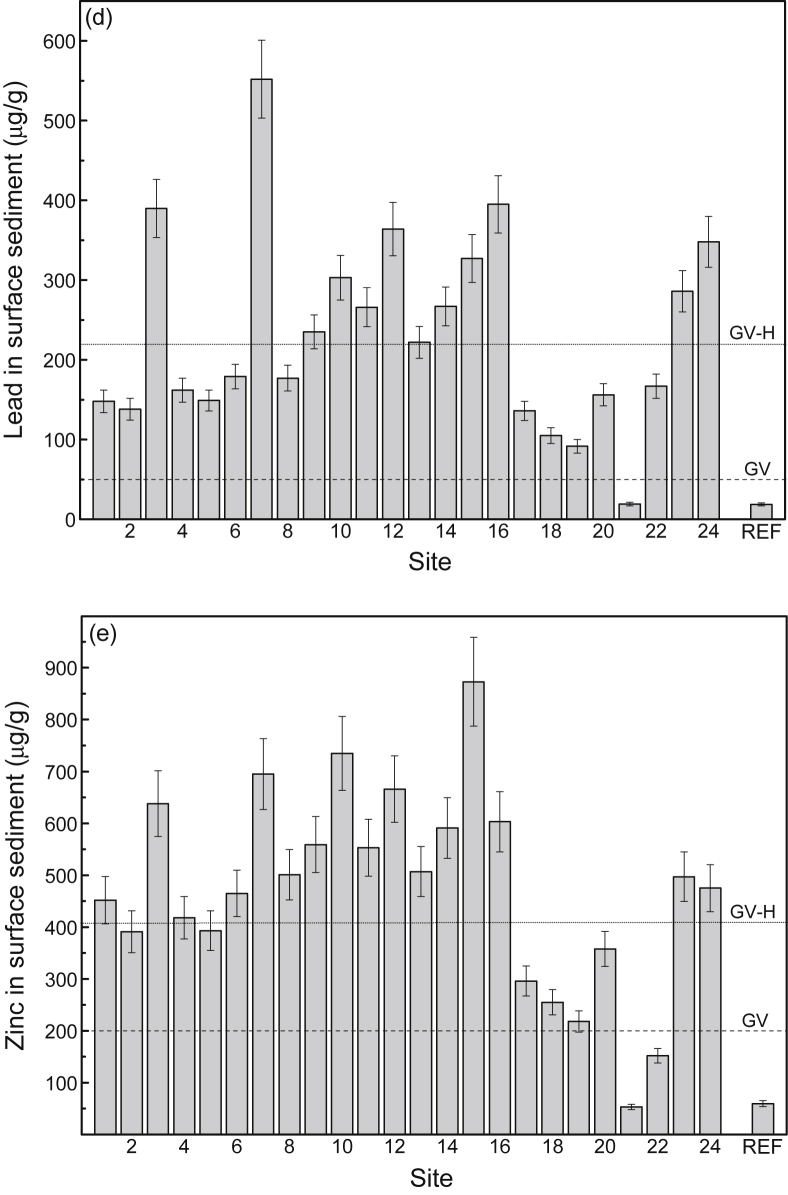
Mean total concentrations (μg/g dry weight) (and 84% confidence intervals; *p ≤* 0.05) of (a) cadmium, (b) chromium, (c) copper, (d) lead and (e) zinc in surface sediment from study sites (1–24) in the Sydney Estuary. The mean metal concentrations for the 15 reference sites (REF) in the Hawkesbury Estuary are shown for comparison. Also shown (horizontal lines) are the metal guideline values (GV and GV-H) for protecting marine biota in Australia [6]. The GV represents a metal concentration below which there is a low potential for ecotoxicological effect, while the GV-H represents a metal concentration above which there is a high potential for ecotoxicological effect. See Fig. 7 for site locations and Table 4 (Hawkesbury Estuary) and 5 (Sydney Estuary) for global positioning system (GPS) coordinates.

The general physico-chemistry of surface waters at 15 study sites in the near-pristine Hawkesbury Estuary is provided in Table 1. The mean percentages of sand, silt and clay (as dry weight) in surface sediment at each site are presented in Fig. 1 (raw data provided in Appendix A, Table S1), while the mean concentrations (% dry weight) of particulate organic carbon (POC), aluminium (Al) and iron (Fe) in surface sediment and suspended particulate matter (SPM) are provided in Fig. 2a and b, respectively (raw data provided in Appendix A, Table S2). The mean concentrations of cadmium (Cd), chromium (Cr), copper (Cu), lead (Pb) and zinc (Zn) in surface water and sediment (combined for all 15 sites) are presented in Fig. 3, Fig. 4, respectively (raw data provided in Appendix A, Tables S3 and S4, respectively). The mean concentrations of Cd, Cr, Cu, Pb and Zn in filtered (<0.2 μm) surface water in the Hawkesbury Estuary, relative to other near-pristine estuarine sites globally, are given in Appendix A (Table S5). The mean concentrations of Cd, Cr, Cu, Pb and Zn in SPM (>0.2 μm), relative to world river average SPM, continental crust and fine sediments (<63 μm) in core samples (at pre-anthropogenic depths) from previous studies in the Hawkesbury Estuary, are provided in Appendix A (Table S6).

**Table 1:** Physico-chemistry of surface water from the Hawkesbury Estuary.

Site^a^	Salinity (%.)	pH	pe	Temperature (^o^C)	Dissolved oxygen (% saturation)	Suspended particulate matter (mg/L)	Dissolved organic carbon (mg/L)^b^	Chlorophyll *a* (μg/L)
A	25.0 ± 1.2^c^	7.54 ± 0.07	6.81 ± 0.08	19.6 ± 2.0	76.2 ± 5.3	5.49 ± 1.41	1.33 ± 0.11	7.1 ± 3.1
B	31.5 ± 0.9	7.84 ± 0.06	6.60 ± 0.07	19.6 ± 2.0	80.1 ± 5.0	2.94 ± 1.05	1.10 ± 0.08	5.0 ± 2.5
C	33.7 ± 0.5	7.99 ± 0.04	6.44 ± 0.05	19.6 ± 1.9	84.0 ± 4.5	3.27 ± 1.08	1.00 ± 0.06	3.9 ± 2.1
D	34.1 ± 0.5	8.03 ± 0.04	6.40 ± 0.05	19.6 ± 1.9	84.8 ± 4.5	2.31 ± 0.90	0.95 ± 0.06	3.7 ± 2.0
R1	27.2 ± 1.1	7.77 ± 0.06	6.71 ± 0.07	19.6 ± 2.0	78.8 ± 5.1	4.78 ± 1.31	1.17 ± 0.10	5.4 ± 2.7
R2	27.3 ± 1.1	7.76 ± 0.06	6.72 ± 0.07	19.6 ± 2.0	78.4 ± 5.2	4.73 ± 1.30	1.18 ± 0.10	5.5 ± 2.7
R3	27.2 ± 1.1	7.69 ± 0.06	6.73 ± 0.07	19.6 ± 2.0	77.8 ± 5.2	4.81 ± 1.32	1.22 ± 0.10	6.3 ± 2.9
R4	27.5 ± 1.0	7.79 ± 0.06	6.72 ± 0.07	19.6 ± 2.0	79.2 ± 5.1	4.66 ± 1.28	1.20 ± 0.10	5.3 ± 2.6
R5	25.5 ± 1.2	7.56 ± 0.06	6.79 ± 0.08	19.6 ± 2.0	74.8 ± 5.5	5.09 ± 1.33	1.31 ± 0.11	7.0 ± 3.1
R6	26.9 ± 1.1	7.74 ± 0.06	6.70 ± 0.07	19.6 ± 2.0	79.7 ± 5.1	4.93 ± 1.34	1.20 ± 0.10	5.5 ± 2.7
R7	28.7 ± 1.0	7.81 ± 0.06	6.65 ± 0.07	19.6 ± 2.0	77.7 ± 5.2	4.61 ± 1.30	1.16 ± 0.09	5.3 ± 2.6
R8	33.2 ± 0.7	7.94 ± 0.05	6.50 ± 0.06	19.6 ± 2.0	81.8 ± 4.9	3.54 ± 1.12	1.06 ± 0.07	4.4 ± 2.3
R9	33.4 ± 0.6	7.97 ± 0.04	6.48 ± 0.05	19.6 ± 2.0	82.9 ± 4.7	2.74 ± 1.01	1.01 ± 0.07	4.1 ± 2.2
R10	32.7 ± 0.7	7.92 ± 0.05	6.53 ± 0.06	19.6 ± 1.9	82.7 ± 4.7	3.41 ± 1.10	1.08 ± 0.07	4.5 ± 2.2
R11	30.6 ± 0.9	7.83 ± 0.06	6.63 ± 0.07	19.6 ± 2.0	77.7 ± 5.2	4.88 ± 1.32	1.13 ± 0.09	5.3 ± 2.6
Mean	29.6 ± 1.0	7.82 ± 0.06	6.63 ± 0.07	19.6 ± 2.0	79.7 ± 5.2	4.15 ± 1.23	1.14 ± 0.10	5.2 ± 2.7

a Study sites are shown in Fig. 7.

b Filtered (<0.2 μm).

c Mean ± 84% confidence limit (i.e. *p* ≤ 0.05). n = 24.

**Fig. 5:**
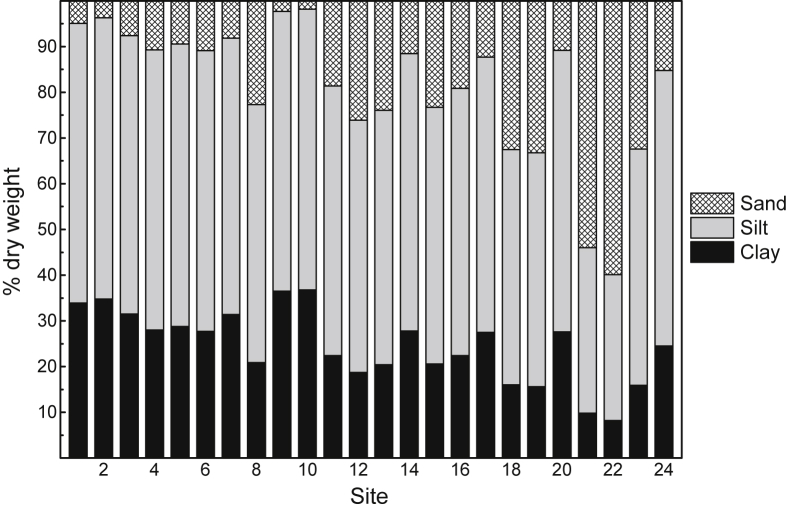
Mean percentage distribution of sand, silt and clay (as dry weight) in surface sediment from study sites in the Sydney Estuary – see Fig. 7 for site locations.

**Fig. 6:**
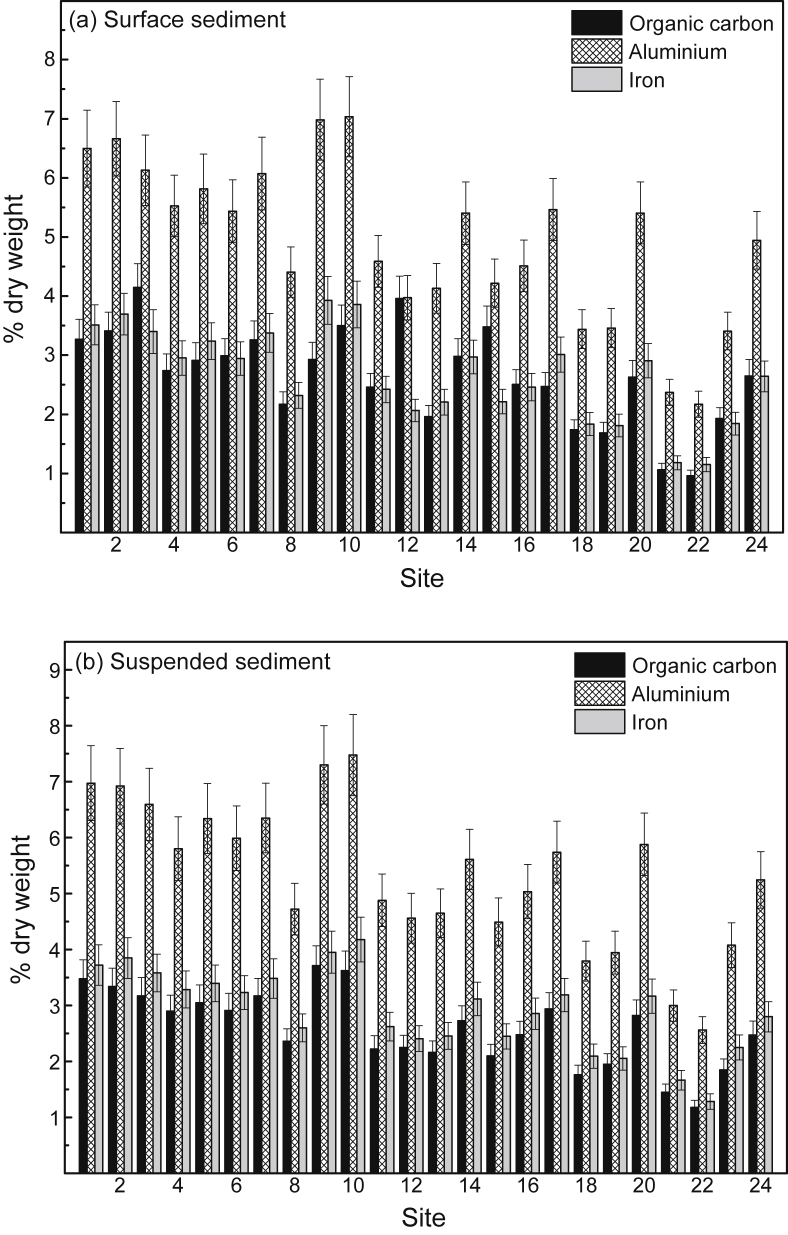
Mean concentrations (% dry weight) (and 84% confidence limits; *p* ≤ 0.05) of particulate organic carbon, aluminium and iron in (a) surface sediment and (b) suspended particulate matter from study sites in the Sydney Estuary – see Fig. 7 for site locations.

The general physico-chemistry of surface waters at 24 study sites in the highly urbanized Sydney Estuary is provided in Table 2. The mean percentages of sand, silt and clay (as dry weight) in surface sediment are presented in Fig. 5 (raw data provided in Appendix A Table S7), while the mean concentrations (% dry weight) of POC, Al and Fe in surface sediment and SPM are provided in Fig. 6a and b, respectively (raw data provided in Appendix A, Table S8). The mean filtered (<0.2 μm) and total (including SPM) concentrations of Cd, Cr, Cu, Pb and Zn in surface water at the 24 study sites (shown as 1–24), relative to national water quality guideline values, are presented in Fig. 3 (raw data provided in Appendix A, Table S3). The mean percentage distribution of Cr (as Cr(III)/Cr(IV)) in filtered (<0.2 μm) surface water, relative to other estuarine and nearshore (oxic) surface waters, is given in Appendix A (Table S9). The partition coefficients (K_d_ as log_10_ L/kg) of Cd, Cr, Cu, Pb and Zn, relative to physico-chemically similar estuarine waters, are provided in Appendix A (Table S10). The mean concentrations of Cd, Cr, Cu, Pb and Zn in surface sediments at the 24 study sites (shown as 1–24), relative to national sediment quality guideline values, are presented in Fig. 4 (raw data provided in Appendix A, Table S4).

**Table 2:** Physico-chemistry of surface water from the Sydney Estuary.

Site^a^	Salinity (‰)	pH	pe	Temperature (^o^C)	Dissolved oxygen (% saturation)	Suspended particulate matter (mg/L)	Dissolved organic carbon (mg/L)^b^	Chlorophyll *a* (μg/L)
1	25.2 ± 1.2^c^	7.53 ± 0.07	6.80 ± 0.08	19.5 ± 2.0	74.8 ± 5.5	7.72 ± 1.75	1.58 ± 0.13	7.9 ± 3.3
2	27.0 ± 1.1	7.60 ± 0.07	6.70 ± 0.07	19.5 ± 2.0	76.8 ± 5.3	7.31 ± 1.65	1.45 ± 0.12	7.5 ± 3.1
3	27.1 ± 1.1	7.65 ± 0.06	6.68 ± 0.07	19.5 ± 2.0	76.2 ± 5.4	7.42 ± 1.65	1.39 ± 0.11	7.4 ± 3.1
4	28.6 ± 1.0	7.76 ± 0.06	6.63 ± 0.07	19.5 ± 2.0	77.9 ± 5.2	6.75 ± 1.54	1.28 ± 0.11	6.2 ± 2.9
5	29.5 ± 1.0	7.82 ± 0.06	6.66 ± 0.07	19.5 ± 2.0	77.2 ± 5.3	6.11 ± 1.45	1.21 ± 0.10	5.5 ± 2.7
6	32.0 ± 0.8	7.84 ± 0.06	6.60 ± 0.07	19.5 ± 2.0	77.4 ± 5.3	4.35 ± 1.28	1.15 ± 0.10	4.9 ± 2.5
7	32.0 ± 0.8	7.83 ± 0.06	6.63 ± 0.07	19.5 ± 2.0	78.4 ± 5.2	4.86 ± 1.34	1.19 ± 0.10	4.8 ± 2.5
8	31.9 ± 0.8	7.87 ± 0.05	6.57 ± 0.06	19.5 ± 2.0	79.3 ± 5.2	5.54 ± 1.43	1.19 ± 0.10	5.0 ± 2.5
9	32.3 ± 0.8	7.97 ± 0.04	6.47 ± 0.05	19.5 ± 2.0	78.2 ± 5.2	4.34 ± 1.29	1.14 ± 0.09	4.6 ± 2.3
10	32.7 ± 0.7	7.97 ± 0.04	6.45 ± 0.05	19.5 ± 2.0	78.3 ± 5.1	4.20 ± 1.27	1.10 ± 0.08	4.3 ± 2.2
11	33.5 ± 0.6	8.01 ± 0.04	6.42 ± 0.05	19.5 ± 2.0	78.9 ± 5.1	3.97 ± 1.19	1.02 ± 0.07	3.9 ± 2.0
12	33.6 ± 0.6	7.98 ± 0.04	6.40 ± 0.05	19.5 ± 2.0	81.1 ± 4.9	3.90 ± 1.16	0.95 ± 0.06	3.7 ± 1.9
13	33.8 ± 0.6	8.04 ± 0.04	6.44 ± 0.05	19.5 ± 2.0	82.2 ± 4.8	3.86 ± 1.13	1.00 ± 0.07	3.6 ± 1.8
14	33.2 ± 0.6	7.97 ± 0.04	6.50 ± 0.05	19.5 ± 2.0	76.0 ± 5.4	4.41 ± 1.28	1.08 ± 0.07	4.4 ± 2.2
15	33.8 ± 0.6	8.03 ± 0.04	6.39 ± 0.05	19.5 ± 2.0	81.4 ± 4.9	3.92 ± 1.16	0.96 ± 0.06	3.5 ± 1.8
16	33.9 ± 0.5	8.04 ± 0.04	6.41 ± 0.05	19.5 ± 2.0	82.2 ± 4.8	3.79 ± 1.09	0.91 ± 0.06	3.3 ± 1.7
17	25.9 ± 1.2	7.51 ± 0.07	6.81 ± 0.08	19.5 ± 2.0	74.1 ± 5.5	7.48 ± 1.78	1.61 ± 0.13	7.8 ± 3.2
18	28.3 ± 1.0	7.69 ± 0.06	6.71 ± 0.07	19.5 ± 2.0	75.2 ± 5.4	6.34 ± 1.45	1.45 ± 0.12	6.4 ± 3.0
19	32.0 ± 0.8	7.92 ± 0.05	6.57 ± 0.07	19.5 ± 2.0	79.9 ± 5.0	5.58 ± 1.39	1.17 ± 0.11	5.1 ± 2.5
20	33.4 ± 0.7	7.97 ± 0.04	6.52 ± 0.06	19.5 ± 2.0	80.2 ± 5.0	4.45 ± 1.27	1.06 ± 0.08	4.0 ± 2.0
21	33.4 ± 0.6	7.98 ± 0.04	6.57 ± 0.06	19.5 ± 2.0	82.9 ± 4.7	4.13 ± 1.20	1.01 ± 0.07	3.9 ± 2.0
22	33.8 ± 0.5	8.00 ± 0.04	6.44 ± 0.05	19.5 ± 2.0	83.2 ± 4.6	3.91 ± 1.08	0.95 ± 0.06	3.7 ± 1.9
23	34.0 ± 0.5	8.04 ± 0.04	6.40 ± 0.05	19.5 ± 1.9	83.0 ± 4.6	4.51 ± 1.20	0.92 ± 0.06	3.3 ± 1.6
24	34.3 ± 0.5	8.08 ± 0.04	6.36 ± 0.05	19.5 ± 1.9	84.2 ± 4.5	4.34 ± 1.10	0.89 ± 0.05	3.1 ± 1.6
Mean	31.4 ± 1.0	7.88 ± 0.05	6.55 ± 0.06	19.5 ± 2.0	79.1 ± 5.2	4.98 ± 1.33	1.14 ± 0.10	4.8 ± 2.5

a Study sites are shown in Fig. 7.

b Filtered (<0.2 μm).

c Mean ± 84% confidence limit (i.e. *p* ≤ 0.05). n = 24.

The mean enrichment factors (EFs) for Cd, Cr, Cu, Pb and Zn in SPM (>0.2 μm) in the Sydney Estuary, relative to physico-chemically similar estuarine waters, are provided in Appendix A (Table S11). The cumulative mean metal EFs for the dissolved/colloidal (<0.2 μm) and SPM phases at each site are provided in Markich and Jeffree [1]. The mean EFs for Cd, Cr, Cu, Pb and Zn in surface sediments from the Sydney Estuary, relative to physico-chemically similar estuarine waters, are provided in Appendix A (Table S12). The cumulative mean metal EFs for surface sediment at each site are provided in Markich and Jeffree [1]. Linear regression fits (r^2^ values) for mean metal EFs between (i) surface sediment and SPM, (ii) surface sediment and filtered (<0.2 μm) water, and (iii) SPM and filtered (<0.2 μm) water, are given in Table 3.

**Table 3:** Linear regression fit (r^2^) matrix between metal enrichment factors in surface sediment, suspended particulate matter (SPM) and filtered (<0.2 μm) surface water^a^.

	Sediment	SPM
**Cadmium**		
SPM	0.97	–
Water	0.91	0.91
**Chromium**		
SPM	0.98	–
Water	0.91	0.93
**Copper**		
SPM	0.96	–
Water	0.92	0.93
**Lead**		
SPM	0.96	–
Water	0.91	0.92
**Zinc**		
SPM	0.96	–
Water	0.88	0.87

a Regressions are highly significant (*p* < 0.01).

The 15 study sites in the Hawkesbury Estuary and the 24 study sites in the Sydney Estuary, are displayed in Fig. 7, with their global positioning system (GPS) coordinates given in Table 4, Table 5, respectively. Linear regression equations and fits (r^2^ values) for Cd, Cr, Cu, Pb and Zn concentrations as a function of Al or Fe concentration in SPM and surface sediment in the Hawkesbury Estuary, are given in Table 6.

**Fig. 7:**
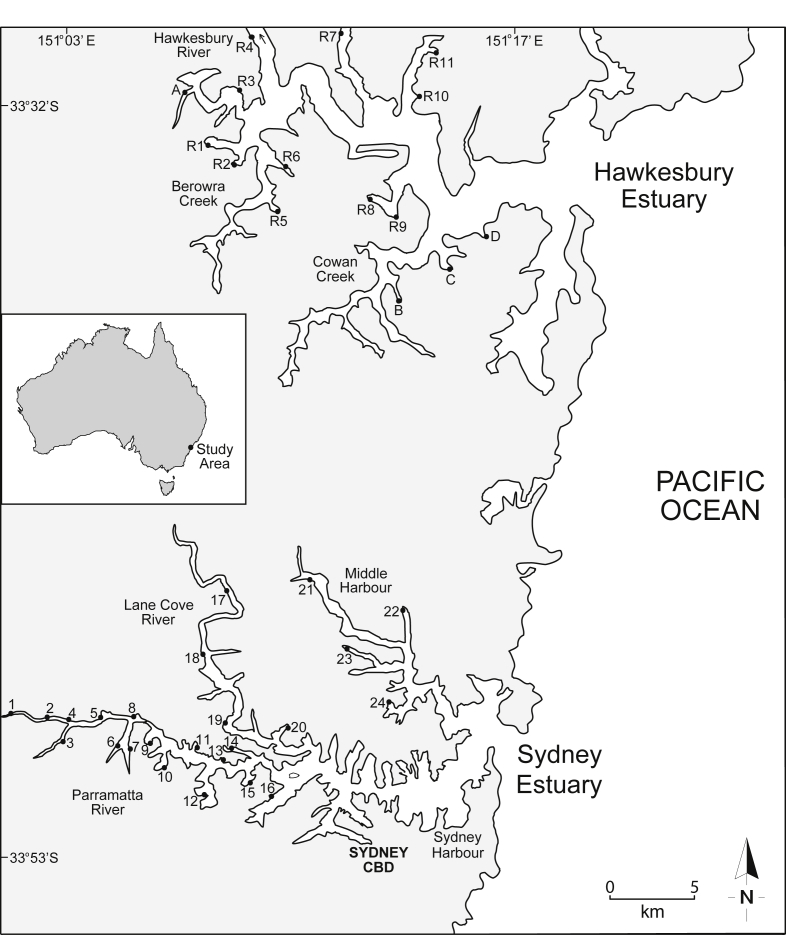
Location map showing the study sites in the Hawkesbury and Sydney estuaries, south-eastern Australia (inset). Of the 15 study sites in the Hawkesbury Estuary, R1–11 are sites where the mussel, *Xenostrobus securis*, was present, and A, B, C and D are sites where mussels were absent (see Markich and Jeffree [1]).

**Table 4:** Study sites in the Hawkesbury Estuary.

Site	Location	GPS coordinates^a^
A	Marramarra Creek (Canoelands)	−33.513958, 151.114472
B	Cowan Creek (Yeomans Bay)	−33.617343, 151.227581
C	Hawkesbury River (Refuge Bay)	−33.604624, 151.256586
D	Hawkesbury River (Little Pittwater Bay)	−33.588744, 151.273071
R1	Berowra Creek (Coba Bay)	−33.542362, 151.123591
R2	Berowra Creek (Donnybrook Bay)	−33.548766, 151.134733
R3	Marramurra Creek (Friendly Bay)	−33.514284, 151.136076
R4	Hawkesbury River (Canoelands)	−33.494638, 151.145603
R5	Berowra Creek (Bujwa Bay)	−33.574523, 151.158707
R6	Berowra Creek (Kimmerikong Bay)	−33.550980, 151.164325
R7	Mooney Mooney Creek (Fox Bay)	−33.489037, 151.192649
R8	Hawkesbury River (Porto Bay)	−33.567611, 151.210078
R9	Hawkesbury River (Porto Bay)	−33.574313, 151.223573
R10	Mullet Creek (Woy Woy)	−33.523199, 151.241256
R11	Mullet Creek (Wondabyne)	−33.498480, 151.258484

a Global positioning system (GPS) coordinates shown as latitude and longitude (as per world geodetic system 84).

**Table 5:** Study sites in the Sydney Estuary.

Site	Location	GPS coordinates^a^
1	Parramatta River (Rangihou Reserve)	−33.815736, 151.015557
2	Parramatta River (Rydalmere)	−33.818624, 151.036464
3	Duck River (Silverwater)	−33.829804, 151.045764
4	Parramatta River (Eric Primrose Reserve)	−33.823122, 151.049218
5	Parramatta River (George Kendall Reserve)	−33.821961, 151.067403
6	Parramatta River (Homebush Bay)	−33.834004, 151.076630
7	Parramatta River (Homebush Bay)	−32.836092, 151.082980
8	Parramatta River (Memorial Park)	−33.819828, 151.085997
9	Parramatta River (Brays Bay)	−33.833625, 151.094738
10	Parramatta River (Majors Bay)	−33.843682, 151.102696
11	Parramatta River (Glades Bay)	−33.835135, 151.120888
12	Parramatta River (Exile Bay)	−33.856989, 151.119385
13	Parramatta River (Bedlam Bay)	−33.840935, 151.133072
14	Tarban Creek (Hunters Hill)	−33.836668, 151.138756
15	Parramatta River (Five Dock Bay)	−33.852053, 151.144346
16	Parramatta River (Iron Cove)	−33.857635, 151.156840
17	Lane Cove River (River Reserve)	−33.793658, 151.157028
18	Lane Cove River (Magdala Park)	−33.806638, 151.142243
19	Lane Cove River (Boronia Park)	−33.826129, 151.143686
20	Lane Cove River (Tambourine Bay)	−33.829222, 151.164001
21	Middle Harbour (Roseville Chase)	−33.770023, 151.202078
22	Middle Harbour (Bantry Bay)	−33.774049, 151.229147
23	Middle Harbour (Sugarloaf Bay)	−33.792422, 151.217658
24	Middle Harbour (Long Bay)	−33.814787, 151.223648

a Global positioning system (GPS) coordinates shown as latitude and longitude (as per world geodetic system 84).

**Table 6:** Linear regression equations and coefficients of determination (r^2^) for cadmium, chromium, copper, lead and zinc concentrations (μg/g dry weight) as a function of aluminium or iron concentration (as % dry weight) for suspended particulate matter and surface sediment in the Hawkesbury Estuary^a^.

Metal	Suspended particulate matter				Surface sediment			
	Aluminium (Al)		Iron (Fe)		Aluminium (Al)		Iron (Fe)	
	Equation	r^2^	Equation	r^2^	Equation	r^2^	Equation	r^2^
Cadmium	0.0112(Al) + 0.0523	0.72	0.0195(Fe) + 0.0556	0.73	0.0102(Al)+ 0.0471	0.72	0.0218(Fe) + 0.0378	0.71
Chromium	4.89(Al) – 1.68	0.97	8.59(Fe) – 0.344	0.98	4.00(Al) + 0.696	0.97	7.24(Fe) + 1.10	0.98
Copper	2.07(Al) + 2.45	0.88	3.65(Fe) + 2.98	0.86	1.91(Al) + 2.79	0.90	3.35(Fe) + 2.88	0.91
Lead	2.85(Al) + 2.78	0.90	4.88(Fe) + 3.88	0.90	2.64(Al) + 2.49	0.92	4.56(Fe) + 3.62	0.93
Zinc	8.73(Al) + 12.2	0.85	15.2(Fe) + 14.9	0.84	8.25(Al)+ 9.91	0.87	15.6(Fe) + 8.38	0.88

a Linear regressions are highly significant (*p* < 0.01).

## 2 Experimental design, materials, and methods

### 2.1 Study area

The Sydney Estuary, comprising the Parramatta and Lane Cove Rivers, and Middle and Sydney Harbours (Fig. 7), is a tide-dominated drowned river valley with a catchment area of 484 km^2^ (∼90% urbanized) and a length of 30 km. Tides are microtidal (mean and maximum tidal range is ∼1.0 m and 2.2 m, respectively) and mixed semi-diurnal. Twenty-four sites (Table 5; Fig. 7) were selected *a priori*, representing a wide range of contamination, where mussels (*Xenostrobus securis*) resided in surface sediments [1]. In addition, 15 near-pristine sites (Table 4; Fig. 7), surrounded by national parks with minimal urban influences, were selected in the lower reaches of the adjacent Hawkesbury Estuary, which shares the same geology as the Sydney Estuary. These sites were used as reference (or background) sites for direct comparison with those in the Sydney Estuary. Surface sediments and water were collected at all sites in both estuaries. Five metals – Cd, Cr, Cu, Pb and Zn – were identified as key chemical contaminants in the Sydney Estuary, based on a comprehensive scoping study [1].

### 2.2 Surface sediment

Sediments were collected at low tide within the intertidal zone in July 2003 and June 2004. At each site, three sediment samples, each a composite of five locations (immediately below where mussels were sampled), were collected using polyethylene containers (500 mL), which were moved through oxic surface (depth <20 mm) sediment until full. Sealed samples were transported to the laboratory (in cool, insulated containers) within 6 h of collection and maintained at 3 °C prior to analysis.

Sediment samples from each site were thoroughly mixed before being wet-sieved (2 mm nylon mesh) using site water, oven-dried (40 °C) to a constant measured weight and homogenised (agate mill). Two sub-samples (0.1 g) from each composite sample were then solubilised in 14.4 M nitric acid (4 mL), 22.6 M hydrofluoric acid (1.5 mL) and 9.8 M hydrogen peroxide (3 mL) using a microwave digestion system (Milestone ETHOS 1). The resulting clear digest solutions were cooled, filtered (0.2 μm polycarbonate; Whatman Nuclepore) and volume adjusted (25 mL) with deionised water (Milli-Q, 18 MΩ/cm) prior to metal analysis.

Dried and homogenised sediment (<2 mm) was treated with 9.8 M hydrogen peroxide (at 70 °C until bleached) to remove organic matter, and sodium pyrophosphate (12 h in an overhead shaker) to promote dispersion. Sediment particle size distribution (0.02–2000 μm) was then determined using laser diffraction (Malvern Mastersizer 2000 with a Hydro G dispersion unit), whereby the weight percentages of clay (0.02–2.0 μm), silt (2.0–63 μm) and sand (63–2000 μm) were calculated. Dried and homogenised sediment (<2 mm) samples (10 mg) from each site were placed in silver capsules within a glass desiccator and treated with vaporous hydrochloric acid (12 M) to remove inorganic carbon, before being dried under vacuum and homogenised (agate mill), prior to particulate organic carbon (POC) analysis (expressed as a percentage of the dry mass of sediment; % POC).

### 2.3 Surface water

Surface water (∼50 cm depth) at each site was measured *in situ* for pH, redox potential (pe), temperature (^o^C), salinity (‰) and dissolved oxygen (% saturation) with a YSI-6000UPG sonde. The pH was measured with a glass combined electrode calibrated using a tris/tris-HCl buffer (on a total pH scale) according to Del Valls and Dickson [2]. All other electrodes/probes were calibrated according to the manufacturer's instructions using appropriate standard solutions. Surface water at each site was also collected by hand from the bow of an electric powered inflatable boat (moving against the tidal flow) using a two-person ‘‘clean’’ handling protocol, according to Markich and Brown [3]. Water was collected, in duplicate, using either 1 L opaque low-density polyethylene (LDPE) bottles (for metals and chlorophyll *a*) or pre-combusted (450 °C, 6 h) 1 L borosilicate (amber) glass bottles (for organic carbon). Surface water was collected, or measured *in situ*, twice a month for 12 months (i.e. 24 sampling events from July 2003 to June 2004), 3 h either side of high tide. All water samples were collected during dry or low rainfall (<5 mm/day) conditions (for at least 96 h prior to sampling) with wind speed <10 km/h. Water samples were transported to the laboratory (on ice in insulated containers) within 8 h of collection and stored at 3 °C.

Within 24 h of collection, a 180 mL sub-sample of water from each 1 L LDPE bottle was filtered (0.2 μm polycarbonate; Whatman Nuclepore) under vacuum into a 100 mL LDPE bottle and acidified (pH < 2 with 1 mL of 14.4 M nitric acid). Removal of the salt matrix and preconcentration of metals was performed using the ammonium pyrrolidine dithiocarbamate solvent extraction/mercury back-extraction procedure described by Batterham and Perry [4] and Batterham et al. [5]. Samples were stored at 3 °C prior to metal analysis. A 120 mL sub-sample of water from each 1 L glass bottle was filtered (0.2 μm polysulfone; Gelman) under vacuum into 40 mL pre-combusted amber glass vials (with an aluminium lined cap) and stored at 3 °C prior to organic carbon analysis. For all samples, the first 10 mL of filtrate was discarded, and the next 20 mL was used to rinse the sample containers (and then discarded). All procedures were performed in an ISO class 5 laminar flow cabinet (Gelman) to minimise contamination. Duplicates were prepared for each sample (i.e. two samples per site, each prepared in duplicate).

The Australian water quality guidelines for protecting marine biota [6] require a knowledge of the concentrations of Cr(III) and Cr(VI) - the two oxidation states of Cr that typically exist in natural surface waters [7]. Using a 25 mL filtered (0.2 μm) sub-sample of water from each site (decanted from the 1 L LDPE bottle), Cr(VI) was complexed with diphenylcarbazide and preconcentrated using the isoamyl alcohol solvent extraction procedure described by Gardner and Comber [8]. Samples were stored at 3 °C prior to analysis.

Chlorophyll *a* was measured as a proxy for pelagic microalgal abundance. Within 24 h of collection, a 250 mL sub-sample of water from each 1 L LDPE bottle was concentrated onto glass fibre filter circles (GF/F; Whatman), folded, blotted dry, placed in screw-cap centrifuge tubes and extracted with 10 mL of *N*,*N*-dimethylformamide (Merck) in the dark at 25 °C for 1 h. Extracts were centrifuged at 24,000 x *g*. Supernatants were decanted into clean centrifuge tubes (constant volume of 10 mL), transferred to a quartz cuvette and scanned with a UV–visible spectrophotometer (Shimadzu UV-2550). Chlorophyll *a* concentrations were calculated using the absorption maxima and specific absorption coefficients provided by William and Paul [9]. Duplicates were prepared for each sample (i.e. two samples per site, each prepared in duplicate).

### 2.4 Suspended particulate matter

Two surface water samples (500 mL) from each site were filtered onto pre-weighed filter circles (0.2 μm polycarbonate; Whatman Nuclepore). At the end of filtration, 80 mL of deionised water (Milli-Q, 18 MΩ/cm) was passed through the filters to wash out residual sea salt. The filters were then oven dried (35 °C) to a constant weight before measuring the mass of particles with an analytical balance (Sartorius). The dry mass concentration of SPM per unit volume of water (mg/L^)^ for each sample was determined from weight measurements on three replicate filters. The filter circles (including blanks) were then solubilised in 14.4 M nitric acid (4 mL), 22.6 M hydrofluoric acid (1.5 mL) and 9.8 M hydrogen peroxide (3 mL) using a microwave digestion system (Milestone ETHOS 1). The resulting clear digest solutions were cooled, filtered (0.2 μm) and volume adjusted (25 mL) with deionised water (Milli-Q, 18 MΩ/cm) prior to metal analysis.

For the determination of suspended POC, water samples were filtered (as described above) using pre-combusted (450 °C for 1 h) membranes, which were later treated with 1 M hydrochloric acid to remove inorganic carbon. The final suspended POC concentration was calculated from the dry mass of organic carbon measured on the sample filter (and from the volume of sample filtered) and expressed as a percentage of the dry mass of SPM (% POC). In these calculations, the correction was made for the average mass of carbon determined for blank filters. Although the % POC does not provide exact information on the total particulate organic matter, it provides a means for comparing samples in terms of approximate contribution of organic matter to SPM.

### 2.5 Metal and organic carbon analyses

The concentrations of Cd, Cu, Pb and Zn in surface water and digest solutions of SPM and sediment were measured using inductively coupled plasma mass spectrometry (ICPMS; HP Agilent 4500). Gallium, indium and rhenium were employed as internal standards to correct for any non-spectral interferences. The concentrations of total Cr, Cr(VI), Al and Fe in surface water and/or SPM and sediment digests were measured using inductively coupled plasma atomic emission spectrometry (Varian Vista AX). The concentration of Cr(III) in surface water was calculated as the difference between total Cr and Cr(VI). Particulate organic carbon was determined by dry combustion and infrared detection of CO_2_ (LECO CNS-2000 Analyzer). Dissolved (<0.2 μm filtered) organic carbon was measured by ultraviolet-persulfate oxidation (Tekmar Dohrmann Pheonix 8000 Analyzer) following standard method 5310c [10].

### 2.6 Quality assurance

All reagents used were analytical grade, except for ultrapure nitric acid (Normaton). All solutions were prepared with deionised water (Milli-Q, 18 MΩ/cm). Collection containers, filters and apparatus were cleaned and prepared for use following the procedures described in detail by Markich and Brown [3].

Procedural blanks were employed throughout sample collection, pre-treatment and analysis to evaluate contamination. Field blanks consisted of pre-analysed deionised water (Milli-Q, 18 MΩ/cm) that were handled and analysed in the same way as the samples. Typical field blank concentrations were: <1 ng/L Cd, 3 ng/L Cr, 5 ng/L Cu, 1 ng/L Pb and 5 ng/L Zn; indicating negligible contamination. All analyses were corrected for blanks. Standard reference materials (SRMs; National Research Council of Canada (NRCC) harbour sediment PACS-2, NRCC seawater CASS-4, United States Geological Survey MAG-1 marine sediment and Hansell Sargasso seawater DSR-Batch3) and sample duplicates were used to evaluate analytical accuracy and precision, respectively. The mean measured concentrations of Cd, Cr, Cu, Pb, Zn and organic carbon in the SRMs were within their certified ranges. For duplicate samples and SRMs, the percentage coefficient of variation was typically 5–10% for metals and 3–5% for dissolved or particulate organic carbon. The recoveries of spiked Cr(VI) in filtered surface water samples ranged from 94 to 109% (mean of 102%).

### 2.7 Normalisation of metal concentrations in sediments and suspended particulate matter

The concentrations of Cd, Cr, Cu, Pb and Zn in SPM and surface sediment at each site were normalised for (i.e. divided by) the Al concentration (e.g. Cd/Al), to account for natural variation in particle size and mineralogy. Based on fitted linear regressions between the concentrations of Cd, Cr, Cu, Pb or Zn and either Al or Fe (Table 6), both conservative reference elements [11], Al was selected in preference to Fe (even though both elements (i) provided identical (r^2^) linear fits (Table 6), and (ii) are strongly related (r^2^ = 0.99)), since it has a high natural abundance in the earth's crust (not typically affected by anthropogenic inputs) and is closely associated with the aluminosilicate fraction, which is the dominant metal-bearing phase in particulate matter. Integral to this approach was establishing geochemical background (linear regression) equations (Table 6) for Cd, Cu, Cr, Pb and Zn using a set of near-pristine SPM and surface sediments (with ample particle size variability from the 15 sites in the Hawkesbury Estuary; Fig. 1).

### 2.8 Data analyses

Linear regression analyses were used to investigate the relationships between metals in surface water (for both the dissolved/colloidal (<0.2 μm filtered) and SPM (>0.2 μm) phases) and sediment. The assumptions of linear regression were tested, and model adequacy was confirmed in all cases using either raw or transformed (log_10_) data. Significance was tested at the *p* = 0.05 level.

To standardise results among the five different metals (Cd, Cr, Cu, Pb and Zn) and three different matrices (i.e. dissolved/colloidal phase, SPM and surface sediment) in the Sydney Estuary (and between other estuaries), an enrichment factor (EF) approach was used to quantify the level of metal contamination, whereby the mean concentration of Cd, Cr, Cu, Pb and Zn in an environmental matrix (e.g. surface sediment) was divided by its mean “background” concentration, pooled from all 15 near-pristine sites in the adjacent Hawkesbury Estuary. For SPM and surface sediment, metal concentrations were normalised for Al concentration to account for varying particle size and mineralogy among study sites (see Section 2.7). For SPM and surface sediment, a metal EF < 1.5 is consistent with natural weathering processes [12] of the underlying lithology (i.e. no anthropogenic enrichment), an EF of 1.5–5.0 indicates minor/moderate enrichment, an EF of 5.0–20 indicates high enrichment, an EF of 20–40 indicates very high enrichment and an EF > 40 indicates extremely high enrichment.
