# Supplementary material for: Physico-chemical and key metal data for surface waters and sediments of the Sydney and Hawkesbury estuaries, Australia
Source: Data Brief. 2019 Jul 19;25:104255. doi: 10.1016/j.dib.2019.104255 (PMC6685680; doi:10.1016/j.dib.2019.104255)
Supplement: Supplementary file 1 [file mmc1.docx]

Physico-chemical and key metal data for surface waters and sediments of the Sydney and Hawkesbury estuaries, Australia

Scott J. Markich ^a, b,^ *, Ross A. Jeffree ^c^

*^a^ Aquatic Solutions International, “Point Break”, North Narrabeen Beach, NSW 2101, Australia*

*^b^ Department of Environmental Sciences, Macquarie University, 12 Wally’s Walk, North Ryde, NSW 2109, Australia*

*^c^ Jeffree Conservation and Research, 45 Casuarina Rd, Alfords Point, NSW 2234, Australia*

# Appendix A. Supplementary data

.

**Table S1**

Mean percentage distribution of clay (0.02–2.0 µm), silt (2.0–63 µm) and sand (63–2000 µm) in surface sediments (as dry weight) from study sites in the Hawkesbury Estuary. n=6.

| Site^a^ | Clay (%) | Silt (%) | Sand (%) |
| --- | --- | --- | --- |
| A | 4.5 | 18.5 | 77.0 |
| B | 2.8 | 12.5 | 84.7 |
| C | 7.0 | 27.0 | 66.0 |
| D | 0.8 | 5.5 | 93.7 |
| R1 | 29.1 | 61.6 | 9.3 |
| R2 | 13.3 | 47.5 | 39.3 |
| R3 | 22.6 | 59.0 | 18.4 |
| R4 | 18.4 | 53.9 | 27.7 |
| R5 | 21.0 | 56.8 | 22.2 |
| R6 | 25.0 | 61.4 | 13.6 |
| R7 | 11.7 | 43.0 | 45.3 |
| R8 | 15.8 | 51.0 | 33.2 |
| R9 | 33.5 | 61.0 | 5.5 |
| R10 | 9.6 | 35.4 | 55.0 |
| R11 | 37.4 | 61.4 | 1.2 |

^a^ Site locations are shown in Fig. 7. Mussels (*Xenostrobus securis*) were present at sites R1–11 and absent from sites A–D.

**Table S2**

Concentrations (% dry weight) of particulate organic carbon, aluminum and iron in surface sediment and suspended particulate matter from study sites in the Hawkesbury Estuary.

| Site^a^ | Surface sediment | | |  | Suspended particulate matter | | |
| --- | --- | --- | --- | --- | --- | --- | --- |
|  | Organic carbon | Aluminium | Iron |  | Organic carbon | Aluminium | Iron |
| A | 0.55 ± 0.06^b^ | 1.58 ± 0.14 | 0.75 ± 0.07 |  | 0.88 ± 0.09 | 1.91 ± 0.18 | 0.95 ± 0.09 |
| B | 0.45 ± 0.05 | 1.24 ± 0.11 | 0.59 ± 0.05 |  | 0.66 ± 0.07 | 1.73 ± 0.18 | 0.82 ± 0.08 |
| C | 0.78 ± 0.08 | 1.94 ± 0.17 | 0.97 ± 0.10 |  | 1.06 ± 0.11 | 2.54 ± 0.24 | 1.22 ± 0.11 |
| D | 0.35 ± 0.04 | 0.80 ± 0.07 | 0.39 ± 0.04 |  | 0.48 ± 0.05 | 1.13 ± 0.11 | 0.50 ± 0.05 |
| R1 | 2.71 ± 0.28 | 5.79 ± 0.49 | 3.14 ± 0.25 |  | 3.12 ± 0.31 | 6.14 ± 0.60 | 3.32 ± 0.31 |
| R2 | 1.44 ± 0.15 | 3.19 ± 0.27 | 1.63 ± 0.14 |  | 1.62 ± 0.16 | 3.62 ± 0.35 | 1.91 ± 0.18 |
| R3 | 2.34 ± 0.23 | 4.89 ± 0.42 | 2.59 ± 0.23 |  | 2.52 ± 0.26 | 5.41 ± 0.53 | 2.99 ± 0.27 |
| R4 | 1.79 ± 0.19 | 3.83 ± 0.32 | 2.00 ± 0.17 |  | 1.93 ± 0.19 | 4.40 ± 0.43 | 2.33 ± 0.22 |
| R5 | 2.02 ± 0.21 | 4.43 ± 0.39 | 2.32 ± 0.20 |  | 2.41 ± 0.23 | 4.89 ± 0.48 | 2.58 ± 0.24 |
| R6 | 2.51 ± 0.25 | 5.12 ± 0.44 | 2.78 ± 0.25 |  | 2.76 ± 0.28 | 5.51 ± 0.53 | 3.08 ± 0.30 |
| R7 | 1.24 ± 0.13 | 2.91 ± 0.25 | 1.52 ± 0.13 |  | 1.76 ± 0.17 | 3.61 ± 0.35 | 1.83 ± 0.18 |
| R8 | 1.73 ± 0.18 | 3.41 ± 0.29 | 1.80 ± 0.16 |  | 2.06 ± 0.20 | 3.98 ± 0.40 | 2.27 ± 0.21 |
| R9 | 3.15 ± 0.30 | 6.26 ± 0.54 | 3.47 ± 0.31 |  | 3.09 ± 0.32 | 6.54 ± 0.63 | 3.50 ± 0.33 |
| R10 | 1.13 ± 0.11 | 2.21 ± 0.17 | 1.16 ± 0.10 |  | 1.27 ± 0.13 | 2.60 ± 0.25 | 1.39 ± 0.12 |
| R11 | 3.29 ± 0.31 | 6.92 ± 0.61 | 3.90 ± 0.32 |  | 3.59 ± 0.37 | 7.19 ± 0.68 | 4.07 ± 0.39 |

^a^ Study sites are shown in Fig. 7. Mussels (*Xenostrobus securis*) were present at sites R1–11 and absent from sites A–D.

^b^ Mean ± 84% confidence limit (i.e. *p* ≤0.05) from samples taken in July 2003 and June 2004 (combined n=6).

**Table S3**

Filtered (<0.2 µm) and total concentrations (ng/L) of cadmium, chromium, copper, lead and zinc in surface waters from study sites in the Sydney Estuary.

| Site^a^ | Cadmium |  |  | Chromium |  |  | Copper |  |  | Lead |  |  | Zinc |  |
| --- | --- | --- | --- | --- | --- | --- | --- | --- | --- | --- | --- | --- | --- | --- |
|  | Filtered | Total |  | Filtered | Total |  | Filtered | Total |  | Filtered | Total |  | Filtered | Total |
| 1 | 9.84 ± 1.77^b^ | 17.9 ± 2.90 |  | 180 ± 46.1 | 1200 ± 232 |  | 789 ± 128 | 1640 ± 240 |  | 34.1 ± 5.55 | 1550 ± 224 |  | 1480 ± 267 | 4930 ± 720 |
| 2 | 11.9 ± 2.10 | 22.5 ± 3.33 |  | 543 ± 158 | 3620 ± 542 |  | 653 ± 101 | 1330 ± 198 |  | 29.7 ± 4.90 | 928 ± 135 |  | 1780 ± 320 | 6140 ± 881 |
| 3 | 39.9 ± 7.18 | 71.3 ± 10.6 |  | 674 ± 195 | 4490 ± 883 |  | 1030 ± 156 | 2100 ± 306 |  | 91.2 ± 15.6 | 3150 ± 449 |  | 2370 ± 410 | 6980 ± 988 |
| 4 | 13.1 ± 2.40 | 23.0 ± 3.40 |  | 434 ± 136 | 3340 ± 440 |  | 631 ± 94.8 | 1210 ± 174 |  | 43.0 ± 7.53 | 1230 ± 175 |  | 1440 ± 252 | 4110 ± 578 |
| 5 | 17.6 ± 3.07 | 25.9 ± 3.80 |  | 290 ± 89.8 | 1810 ± 371 |  | 515 ± 82.3 | 972 ± 141 |  | 31.1 ± 5.40 | 915 ± 131 |  | 1340 ± 239 | 3620 ± 502 |
| 6 | 25.4 ± 4.70 | 37.4 ± 5.41 |  | 202 ± 65.7 | 1010 ± 155 |  | 680 ± 105 | 1100 ± 175 |  | 43.7 ± 7.49 | 795 ± 115 |  | 1980 ± 360 | 4040 ± 568 |
| 7 | 39.2 ± 7.10 | 62.7 ± 9.17 |  | 280 ± 90.2 | 1120 ± 168 |  | 1070 ± 155 | 1850 ± 266 |  | 110 ± 19.1 | 2620 ± 367 |  | 2760 ± 493 | 6130 ± 840 |
| 8 | 8.33 ± 1.51 | 12.1 ± 1.75 |  | 194 ± 61.5 | 808 ± 121 |  | 699 ± 105 | 1290 ± 189 |  | 52.8 ± 8.79 | 1080 ± 153 |  | 2500 ± 452 | 5320 ± 752 |
| 9 | 7.86 ± 1.39 | 12.5 ± 1.88 |  | 245 ± 79.8 | 1340 ± 193 |  | 621 ± 99.0 | 1130 ± 157 |  | 45.2 ± 7.75 | 1100 ± 158 |  | 2100 ± 391 | 4470 ± 642 |
| 10 | 40.0 ± 6.84 | 58.8 ± 8.98 |  | 385 ± 128 | 1610 ± 238 |  | 648 ± 102 | 1080 ± 158 |  | 58.0 ± 10.1 | 1120 ± 160 |  | 2190 ± 400 | 5090 ± 717 |
| 11 | 14.0 ± 2.62 | 19.4 ± 2.87 |  | 252 ± 82.1 | 933 ± 141 |  | 990 ± 152 | 1620 ± 239 |  | 57.9 ± 10.2 | 1260 ± 176 |  | 2720 ± 491 | 4950 ± 698 |
| 12 | 13.1 ± 2.30 | 19.5 ± 2.97 |  | 198 ± 66.3 | 683 ± 101 |  | 2640 ± 419 | 4260 ± 599 |  | 101 ± 17.3 | 1800 ± 257 |  | 3170 ± 568 | 5980 ± 851 |
| 13 | 6.00 ± 1.04 | 7.18 ± 1.07 |  | 221 ± 92.7 | 737 ± 107 |  | 1160 ± 160 | 1810 ± 252 |  | 69.5 ± 12.2 | 1290 ± 184 |  | 2240 ± 498 | 4480 ± 633 |
| 14 | 22.2 ± 4.10 | 31.7 ± 4.68 |  | 256 ± 80.5 | 883 ± 135 |  | 791 ± 121 | 1410 ± 201 |  | 71.8 ± 12.3 | 1470 ± 210 |  | 2020 ± 359 | 4390 ± 621 |
| 15 | 27.3 ± 5.17 | 37.9 ± 5.69 |  | 354 ± 115 | 1110 ± 162 |  | 1730 ± 256 | 2750 ± 384 |  | 82.7 ± 15.0 | 1480 ± 207 |  | 3880 ± 690 | 7190 ± 997 |
| 16 | 20.6 ± 3.58 | 29.0 ± 4.47 |  | 276 ± 91.8 | 812 ± 117 |  | 1490 ± 220 | 2480 ± 355 |  | 98.0 ± 16.8 | 1920 ± 269 |  | 2700 ± 483 | 5290 ± 738 |
| 17 | 10.1 ± 1.90 | 14.9 ± 2.25 |  | 149 ± 39.9 | 497 ± 92.6 |  | 734 ± 111 | 1530 ± 221 |  | 37.3 ± 6.26 | 1170 ± 164 |  | 1320 ± 232 | 3770 ± 519 |
| 18 | 6.84 ± 1.30 | 9.00 ± 1.49 |  | 162 ± 46.0 | 477 ± 71.0 |  | 274 ± 47.1 | 435 ± 61.8 |  | 35.9 ± 6.10 | 718 ± 102 |  | 1960 ± 308 | 4110 ± 591 |
| 19 | 5.89 ± 1.10 | 8.41 ± 1.27 |  | 157 ± 45.8 | 449 ± 67.5 |  | 707 ± 116 | 1240 ± 185 |  | 29.9 ± 5.12 | 467 ± 67.1 |  | 1800 ± 312 | 3460 ± 484 |
| 20 | 5.73 ± 1.10 | 8.07 ± 1.24 |  | 184 ± 53.8 | 472 ± 69.3 |  | 759 ± 121 | 1220 ± 181 |  | 44.4 ± 7.76 | 854 ± 122 |  | 1670 ± 298 | 3340 ± 448 |
| 21 | 4.63 ± 0.88 | 5.86 ± 0.98 |  | 135 ± 43.0 | 265 ± 47.2 |  | 328 ± 39.0 | 449 ± 62.3 |  | 13.5 ± 2.34 | 176 ± 24.9 |  | 384 ± 65.1 | 817 ± 109 |
| 22 | 28.3 ± 5.06 | 37.2 ± 5.73 |  | 138 ± 42.7 | 276 ± 46.8 |  | 485 ± 59.3 | 674 ± 99.1 |  | 86.7 ± 15.2 | 1110 ± 157 |  | 1920 ± 345 | 3490 ± 494 |
| 23 | 8.10 ± 1.48 | 10.7 ± 1.63 |  | 562 ± 177 | 1810 ± 272 |  | 2130 ± 312 | 3490 ± 489 |  | 97.1 ± 17.1 | 1570 ± 221 |  | 3240 ± 548 | 5890 ± 811 |
| 24 | 5.53 ± 1.05 | 7.19 ± 1.19 |  | 143 ± 44.8 | 421 ± 62.7 |  | 973 ± 139 | 1740 ± 245 |  | 82.1 ± 13.8 | 1790 ± 258 |  | 2500 ± 448 | 4630 ± 638 |
| REF^c^ | 3.95 ± 0.70 | 5.06 ± 0.82 |  | 112 ± 35.0 | 261 ± 39.4 |  | 271 ± 28.9 | 387 ± 56.1 |  | 11.5 ± 1.35 | 148 ± 20.9 |  | 315 ± 49.2 | 656 ± 87.3 |

^a^ Study sites are shown in Fig. 7.

^b^ Mean ± 84% confidence limit (i.e. *p* ≤0.05) from samples taken from July 2003 to June 2004 (n=48).

^c^ REF is the combined mean of all 15 reference sites in the Hawkesbury Estuary (see Fig. 7 and Table 4).

**Table S4**

Total concentrations (µg/g dry weight) of cadmium, chromium, copper, lead and zinc in surface sediments from study sites in the Sydney Estuary.

| Site^a^ | Cadmium | Chromium | Copper | Lead | Zinc |
| --- | --- | --- | --- | --- | --- |
| 1 | 0.96 ± 0.09^b^ | 97.1 ± 8.68 | 93.7 ± 9.02 | 148 ± 14.0 | 452 ± 45.5 |
| 2 | 1.26 ± 0.12 | 298 ± 26.4 | 81.9 ± 7.78 | 138 ± 13.6 | 391 ± 40.2 |
| 3 | 3.48 ± 0.32 | 378 ± 33.4 | 123 ± 11.3 | 390 ± 36.5 | 638 ± 63.2 |
| 4 | 1.31 ± 0.12 | 200 ± 17.6 | 91.0 ± 8.87 | 162 ± 15.0 | 418 ± 40.8 |
| 5 | 0.93 ± 0.09 | 194 ± 17.3 | 85.2 ± 8.22 | 149 ± 13.2 | 393 ± 38.4 |
| 6 | 1.85 ± 0.16 | 104 ± 9.04 | 92.0 ± 8.61 | 179 ± 15.4 | 465 ± 44.9 |
| 7 | 3.25 ± 0.30 | 126 ± 10.3 | 149 ± 13.2 | 552 ± 48.9 | 695 ± 68.2 |
| 8 | 0.63 ± 0.06 | 87.6 ± 7.65 | 103 ± 9.36 | 177 ± 16.2 | 501 ± 48.6 |
| 9 | 0.93 ± 0.08 | 172 ± 15.1 | 132 ± 11.2 | 235 ± 21.3 | 559 ± 54.1 |
| 10 | 3.65 ± 0.33 | 218 ± 18.8 | 140 ± 12.3 | 303 ± 27.9 | 735 ± 71.3 |
| 11 | 0.96 ± 0.09 | 119 ± 9.82 | 159 ± 14.8 | 266 ± 24.4 | 553 ± 54.8 |
| 12 | 1.03 ± 0.09 | 91.2 ± 7.50 | 308 ± 26.4 | 364 ± 33.5 | 666 ± 64.2 |
| 13 | 0.53 ± 0.05 | 86.7 ± 7.11 | 116 ± 10.2 | 222 ± 19.9 | 507 ± 48.2 |
| 14 | 1.68 ± 0.15 | 93.1 ± 7.60 | 142 ± 12.9 | 267 ± 24.2 | 591 ± 58.2 |
| 15 | 1.94 ± 0.18 | 139 ± 11.4 | 186 ± 17.8 | 327 ± 30.1 | 873 ± 85.5 |
| 16 | 1.87 ± 0.17 | 121 ± 10.5 | 235 ± 21.8 | 395 ± 35.9 | 603 ± 58.3 |
| 17 | 0.49 ± 0.04 | 32.1 ± 2.81 | 91.2 ± 8.71 | 136 ± 11.9 | 296 ± 28.9 |
| 18 | 0.30 ± 0.03 | 30.4 ± 2.70 | 77.8 ± 7.59 | 105 ± 9.80 | 255 ± 24.3 |
| 19 | 0.33 ± 0.03 | 28.3 ± 2.53 | 73.1 ± 7.06 | 91.5 ± 8.65 | 218 ± 20.3 |
| 20 | 0.43 ± 0.04 | 47.0 ± 4.20 | 116 ± 10.3 | 156 ± 13.9 | 358 ± 33.8 |
| 21 | 0.10 ± 0.01 | 22.4 ± 2.20 | 13.4 ± 1.45 | 19.0 ± 2.30 | 53.3 ± 5.32 |
| 22 | 1.09 ± 0.10 | 23.8 ± 2.31 | 31.5 ± 3.01 | 167 ± 15.1 | 152 ± 14.3 |
| 23 | 0.48 ± 0.05 | 182 ± 16.2 | 233 ± 21.1 | 286 ± 25.9 | 497 ± 47.6 |
| 24 | 0.29 ± 0.03 | 37.4 ± 3.45 | 159 ± 14.5 | 348 ± 31.9 | 475 ± 45.5 |
| REF^c^ | 0.11 ± 0.01 | 25.2 ± 2.50 | 13.6 ± 1.38 | 18.6 ± 1.95 | 59.6 ± 5.89 |

^a^ Study sites are shown in Fig. 7.

^a^ Mean ± 84% confidence limit (i.e. *p* ≤0.05) from samples taken in July 2003 and June 2004 (combined n=6).

^b^ REF is the combined mean of all 15 reference sites in the Hawkesbury Estuary (see Fig. 7 and Table 4).

**Table S5**

Mean concentrations (ng/L) of cadmium, chromium, copper, lead and zinc in filtered (<0.2–0.4 µm) estuarine surface waters (salinity 20–35‰ and suspended particulate matter <15 mg/L) from near-pristine sites.

| Estuary | Cadmium | Chromium | Copper | Lead | Zinc | Reference |
| --- | --- | --- | --- | --- | --- | --- |
| Bang Pakong (Thailand) | 11.6 | ― | 318 | 17.6 | 230 | [1] |
| Bathurst (Australia) | 4.50 | ― | 191 | ― | 392 | [2] |
| Darwin (Australia) | 5.80 | ― | 281 | 8.63 | 132 | [3] |
| Galveston Bay (United States) | 5.40 | ― | 317 | 7.92 | 166 | [4] |
| Hawkesbury (Australia) | 3.95 | 112 | 271 | 11.5 | 315 | This study |
| Indian (United States) | 3.52 | 121 | 326 | 12.6 | 143 | [5] |
| Krka (Croatia) | 7.53 | ― | 203 | 14.5 | 294 | [6] |
| Ochlockonee (United States) | 4.05 | ― | 292 | ― | ― | [7] |
| Penzé (France) | 11.3 | ― | 187 | 12.2 | ― | [8] |
| Sandon (Australia) | 3.54 | 102 | 217 | 8.95 | 241 | [9] |
| Tay (United Kingdom) | ― | ― | 326 | 16.4 | 408 | [10] |
| Thale Sap (Thailand) | 9.78 | ― | 358 | 13.2 | 346 | [11] |
| Wangquan (China) | 8.61 | ― | 254 | 8.29 | ― | [12] |
| **Mean** | **6.04** | **111** | **266** | **11.6** | **248** |  |

**Table S6**

Concentrations (µg/g dry weight) of cadmium, chromium, copper, lead and zinc in suspended particulate matter (SPM) from near-pristine sites in the Hawkesbury Estuary (20–35‰ salinity), relative to the Sydney Estuary, the world river average, and fine particulate matter from the upper continental crust and sediment (<63 µm) cores (at pre-anthropogenic depth) from the Hawkesbury Estuary.

| Metal | Continental crust^a^ | Hawkesbury Estuary | |  | Sydney Estuary | World river average |
| --- | --- | --- | --- | --- | --- | --- |
|  |  | Sediment (<63 µm)^b^ | SPM^c^ |  | SPM^c^ | SPM^d^ |
| Cadmium | 0.11 ± 0.04^e^ | 0.10 ± 0.02 | 0.12 ± 0.02 |  | 1.58 (4.35)^f^ | 1.55 |
| Chromium | 53 ± 11 | 26 ± 2.4 | 28 ± 3.1 |  | 206 (588) | 130 |
| Copper | 20 ± 7.7 | 14 ± 1.1 | 15 ± 2.5 |  | 161 (441) | 76 |
| Lead | 16 ± 5.1 | 22 ± 4.1 | 20 ± 3.6 |  | 265 (538) | 61 |
| Zinc | 72 ± 11 | 65 ± 8.7 | 65 ± 8.5 |  | 603 (1070) | 208 |

^a^ Gaschnig et al. [13].

^b^ Birch et al. [14], Olmos and Birch [15] and Matthai et al. [16].

^c^ This study.

^d^ Viers et al. [17].

^e^ Mean ± standard deviation.

^f^  Maximum value.

**Table S7**

Mean percentage distribution of clay (0.02–2.0 µm), silt (2.0–63 µm) and sand (63–2000 µm) in surface sediments (as dry weight) from study sites in the Sydney Estuary. n=6.

| Site^a^ | Clay (%) | Silt (%) | Sand (%) |
| --- | --- | --- | --- |
| 1 | 33.9 | 61.2 | 4.9 |
| 2 | 34.8 | 61.5 | 3.7 |
| 3 | 31.5 | 60.9 | 7.6 |
| 4 | 28.0 | 61.3 | 10.7 |
| 5 | 28.8 | 61.8 | 9.4 |
| 6 | 27.7 | 61.4 | 10.9 |
| 7 | 31.4 | 60.5 | 8.1 |
| 8 | 20.9 | 56.4 | 22.7 |
| 9 | 36.5 | 61.2 | 2.3 |
| 10 | 36.8 | 61.4 | 1.8 |
| 11 | 22.4 | 59.0 | 18.6 |
| 12 | 18.7 | 55.2 | 26.1 |
| 13 | 20.4 | 55.7 | 23.9 |
| 14 | 27.8 | 60.7 | 11.5 |
| 15 | 20.6 | 56.1 | 23.3 |
| 16 | 22.4 | 58.5 | 19.1 |
| 17 | 27.5 | 60.2 | 12.3 |
| 18 | 16.0 | 51.5 | 32.5 |
| 19 | 15.6 | 51.2 | 33.2 |
| 20 | 27.6 | 61.6 | 10.8 |
| 21 | 9.8 | 36.2 | 54.0 |
| 22 | 8.2 | 31.9 | 59.9 |
| 23 | 15.9 | 51.7 | 32.4 |
| 24 | 24.5 | 60.3 | 15.2 |

^a^ Site locations are shown in Fig. 7.

**Table S8**

Concentrations (% dry weight) of particulate organic carbon, aluminum and iron in surface sediment and suspended particulate matter from study sites in the Sydney Estuary.

| Site^a^ | Surface sediment | | |  | Suspended particulate matter | | |
| --- | --- | --- | --- | --- | --- | --- | --- |
|  | Organic carbon | Aluminium | Iron |  | Organic carbon | Aluminium | Iron |
| 1 | 3.27 ± 0.34^b^ | 6.50 ± 0.65 | 3.51 ± 0.34 |  | 3.48 ± 0.34 | 6.97 ± 0.67 | 3.72 ± 0.36 |
| 2 | 3.41 ± 0.32 | 6.66 ± 0.63 | 3.70 ± 0.35 |  | 3.34 ± 0.33 | 6.92 ± 0.67 | 3.85 ± 0.36 |
| 3 | 4.15 ± 0.40 | 6.13 ± 0.60 | 3.40 ± 0.37 |  | 3.17 ± 0.33 | 6.59 ± 0.65 | 3.58 ± 0.34 |
| 4 | 2.74 ± 0.28 | 5.53 ± 0.52 | 2.95 ± 0.29 |  | 2.90 ± 0.28 | 5.80 ± 0.57 | 3.29 ± 0.33 |
| 5 | 2.91 ± 0.30 | 5.81 ± 0.59 | 3.24 ± 0.31 |  | 3.05 ± 0.32 | 6.34 ± 0.62 | 3.40 ± 0.33 |
| 6 | 2.99 ± 0.29 | 5.44 ± 0.53 | 2.94 ± 0.29 |  | 2.91 ± 0.31 | 5.99 ± 0.58 | 3.23 ± 0.30 |
| 7 | 3.26 ± 0.32 | 6.07 ± 0.61 | 3.38 ± 0.33 |  | 3.17 ± 0.31 | 6.35 ± 0.62 | 3.48 ± 0.35 |
| 8 | 2.17 ± 0.21 | 4.41 ± 0.43 | 2.32 ± 0.22 |  | 2.36 ± 0.22 | 4.72 ± 0.46 | 2.60 ± 0.25 |
| 9 | 2.93 ± 0.29 | 6.98 ± 0.69 | 3.93 ± 0.41 |  | 3.71 ± 0.36 | 7.30 ± 0.70 | 3.95 ± 0.37 |
| 10 | 3.50 ± 0.35 | 7.04 ± 0.68 | 3.86 ± 0.39 |  | 3.62 ± 0.35 | 7.48 ± 0.73 | 4.18 ± 0.40 |
| 11 | 2.46 ± 0.23 | 4.59 ± 0.44 | 2.42 ± 0.22 |  | 2.22 ± 0.24 | 4.88 ± 0.47 | 2.62 ± 0.27 |
| 12 | 3.96 ± 0.38 | 3.97 ± 0.38 | 2.07 ± 0.19 |  | 2.25 ± 0.22 | 4.56 ± 0.44 | 2.41 ± 0.23 |
| 13 | 1.96 ± 0.19 | 4.13 ± 0.42 | 2.21 ± 0.22 |  | 2.16 ± 0.21 | 4.65 ± 0.43 | 2.46 ± 0.24 |
| 14 | 2.98 ± 0.30 | 5.40 ± 0.53 | 2.97 ± 0.29 |  | 2.73 ± 0.26 | 5.61 ± 0.54 | 3.12 ± 0.30 |
| 15 | 3.48 ± 0.35 | 4.22 ± 0.41 | 2.22 ± 0.21 |  | 2.10 ± 0.21 | 4.49 ± 0.43 | 2.45 ± 0.23 |
| 16 | 2.51 ± 0.25 | 4.51 ± 0.44 | 2.46 ± 0.23 |  | 2.48 ± 0.24 | 5.04 ± 0.49 | 2.85 ± 0.28 |
| 17 | 2.47 ± 0.24 | 5.46 ± 0.52 | 3.01 ± 0.30 |  | 2.94 ± 0.29 | 5.74 ± 0.55 | 3.19 ± 0.30 |
| 18 | 1.74 ± 0.17 | 3.44 ± 0.33 | 1.84 ± 0.20 |  | 1.76 ± 0.17 | 3.79 ± 0.36 | 2.10 ± 0.22 |
| 19 | 1.69 ± 0.18 | 3.46 ± 0.33 | 1.81 ± 0.19 |  | 1.95 ± 0.19 | 3.95 ± 0.38 | 2.05 ± 0.21 |
| 20 | 2.63 ± 0.28 | 5.41 ± 0.52 | 2.91 ± 0.29 |  | 2.82 ± 0.28 | 5.88 ± 0.56 | 3.17 ± 0.31 |
| 21 | 1.06 ± 0.11 | 2.37 ± 0.22 | 1.18 ± 0.12 |  | 1.45 ± 0.15 | 3.00 ± 0.28 | 1.67 ± 0.18 |
| 22 | 0.96 ± 0.10 | 2.17 ± 0.22 | 1.15 ± 0.12 |  | 1.18 ± 0.13 | 2.56 ± 0.24 | 1.28 ± 0.14 |
| 23 | 1.93 ± 0.19 | 3.41 ± 0.32 | 1.84 ± 0.19 |  | 1.85 ± 0.19 | 4.08 ± 0.40 | 2.25 ± 0.22 |
| 24 | 2.65 ± 0.28 | 4.94 ± 0.49 | 2.64 ± 0.26 |  | 2.47 ± 0.25 | 5.24 ± 0.50 | 2.80 ± 0.27 |

^a^ Study sites are shown in Fig. 7.

^b^ Mean ± 84% confidence limit (i.e. *p* ≤0.05) from samples taken in July 2003 and June 2004 (combined n=6).

**Table S9**

Mean percentage distribution of chromium (Cr) in filtered (<0.2–0.4 µm) estuarine and nearshore (oxic) surface waters (salinity 20–35‰).

| Location | Cr(III) | Cr(VI) | Reference |
| --- | --- | --- | --- |
| Abu Kir Bay (Egypt) | 13 | 87 | [18] |
| Columbia Estuary (United States) | 12 | 88 | [19] |
| El-Manzalah Lagoon (Egypt) | 37 | 63 | [20] |
| Georges Estuary (Australia) | 24 | 76 | [21] |
|  | 29 | 71 | [22] |
| Guanabara Bay (Brazil) | 27 | 73 | [23] |
| Halifax Harbour (Canada) | 32 | 68 | [24] |
| Humber Estuary (United Kingdom) | 24 | 76 | [25] |
| Mersey Estuary (United Kingdom) | 38 | 62 | [26] |
| San Francisco Bay (United States) | 11 | 89 | [27] |
| Sydney Estuary (Australia) | 26 | 74 | This study |
|  | 24 | 76 | [22] |
| **Mean** | **25** | **75** |  |

**Table S10**

Mean distribution coefficients (K_d_) for cadmium, chromium, copper, lead and zinc in estuarine surface waters (20–35‰ salinity and <15 mg/L suspended particulate matter)^a^.

| Estuary | Cadmium | Chromium | Copper | Lead | Zinc | Ranking | Reference |
| --- | --- | --- | --- | --- | --- | --- | --- |
| Conwy (United Kingdom) | ― | ― | 5.2 | ― | 5.1 | Cu > Zn | [28] |
| Danshuei (Taiwan) | 4.6 | ― | 5.2 | 6.2 | ― | Pb > Cu > Cd | [29] |
| Darwin/Bynoe (Australia) | 5.1 | ― | 5.0 | 6.6 | 5.6 | Pb > Zn > Cd > Cu | [3] |
| East Hainan (China) | 4.7 | ― | 5.0 | 6.3 | ― | Pb > Cu > Cd | [12] |
| Galveston (United States) | 4.7 | ― | 4.9 | 6.2 | 5.2 | Pb > Zn > Cu > Cd | [4,30] |
| Krka (Croatia) | 5.0 | ― | 5.3 | 6.4 | 5.5 | Pb > Zn > Cu > Cd | [6] |
| Sagami (Japan) | 5.0 | ― | 5.2 | ― | ― | Cu > Cd | [31] |
| Savannah (United States) | 4.8 | ― | 4.9 | ― | 5.1 | Zn > Cu > Cd | [32] |
| Sydney (Australia) | 4.9 | 5.7 | 5.1 | 6.5 | 5.4 | Pb > Cr > Zn > Cu > Cd | This study |
|  | ― | ― | 4.8^b^ | ― | 5.0^b^ | Zn > Cu | [33] |
| Yangtze (China) | 4.7 | ― | ― | ― | 5.1 | Zn > Cd | [34] |
| **Mean** | **4.8** | **5.7** | **5.1** | **6.4** | **5.3** | **Pb > Cr > Zn > Cu > Cd** |  |

^a^ K_d_ values (log_10_ L/kg) describe the distribution ratio between the concentration (µg/kg) of a metal in suspended particulate matter and the concentration (µg/L) of a metal in filtered (<0.2–0.4 µm) surface water (i.e. dissolved/colloidal phase).

^b^ The lower K_d_ values, relative to the present study (and other studies) are most likely due to a weak acid (hydroxylamine hydrochloride/acetic acid) extraction being used, rather than a strong acid extraction.

**Table S11**

Mean enrichment factors for cadmium, chromium, copper, lead and zinc in suspended particulate matter (SPM) from estuaries (salinity 20–35‰; SPM typically <15 mg/L) impacted by anthropogenic input.

| Estuary | Cadmium | Chromium | Copper | Lead | Zinc | Reference |
| --- | --- | --- | --- | --- | --- | --- |
| Guadiana (Portugal) | 4.6 (10)^a^ | ― | 8.5 (23) | 4.5 (11) | 5.1 (18) | [35] |
| Guanabara Bay (Brazil) | 18 | 1.7 | ― | 5.3 | 6.4 | [36] |
| Kali (India) | ― | 2.3 (2.5) | 1.5 (1.6) | ― | 1.3 (1.8) | [37] |
| Mandovi (India) | ― | 14 (30) | 3.3 (5.0) | 4.3 (7.1) | 2.7 (5.1) | [38] |
| Sydney (Australia) | 10 (25)^b^ | 5.9 (14) | 8.0 (24) | 11 (21) | 8.0 (16) | This study |
|  | ― | 2.7 (5.4) | 4.6 (13) | 3.6 (6.6) | 5.2 (12) | [33] |
| Ulla (Spain) | 1.1 (3.2) | 3.2 (4.8) | 6.4 (22) | 4.3 (12) | 2.2 (4.4) | [39] |
| **Mean** | **5.5** | **3.8** | **4.6** | **5.1** | **3.8** |  |

^a^ Mean (and maximum) values, based on iron- or aluminium-normalised SPM concentrations (µg/g dry weight).

^b^ Enrichment factors were calculated by dividing the aluminium-normalised metal SPM concentration (µg/g dry weight) from the Sydney Estuary by that from the Hawkesbury Estuary.

**Table S12**

Mean enrichment factors for cadmium, chromium, copper, lead and zinc in surface sediments from estuaries (salinity 20–35‰) impacted by anthropogenic input^a^.

| Estuary | Cadmium | Chromium | Copper | Lead | Zinc | Reference |
| --- | --- | --- | --- | --- | --- | --- |
| Amarizimnyama (South Africa) | 10 (17)^a^ | 4.4 (7.8) | 3.2 (5.5) | 2.7 (4.9) | 4.3 (7.4) | [40] |
| Brisbane (Australia) | 1.9 (6.9) | ― | 2.4 (4.5) | 3.2 (9.8) | 3.8 (17) | [41] |
| Clyde (United Kingdom) | 2.0 (2.7) | 4.3 (9.0) | 5.0 (12) | 10 (16) | 4.5 (7.0) | [42,43] |
| Estero Salado (Ecuador) | 5.7 (11) | ― | 8.0 (11) | 3.7 (5.1) | 3.0 (3.7) | [44] |
| Forcados (Nigeria) | 20 (33) | 1.6 (2.4) | ― | 8.4 (13) | ― | [45] |
| Ganges (India) | 6.3 (7.8) | 7.0 (7.5) | 12 (12) | 3.6 (4.3) | 3.1 (3.3) | [46] |
| Haraz (Iran) | 28 (51) | ― | 2.0 (2.2) | 6.4 (7.4) | 3.0 (3.9) | [47] |
| Humber (United Kingdom) | ― | 2.2 (3.2) | 4.1 (6.2) | 7.7 (23) | 3.7 (12) | [48] |
| Linggi (Malaysia) | 4.2 (7.1) | ― | ― | 3.6 (6.0) | 2.8 (3.2) | [49] |
| Loire (France) | 3.5 (16) | 1.3 (2.8) | 1.4 (6.9) | 3.0 (7.2) | 2.1 (6.3) | [50] |
| Mangonbangon (Philippines) | ― | 2.2 (3.2) | 5.7 (8.3) | ― | 5.4 (6.9) | [51] |
| Matanza (Argentina) | 1.9 (2.1) | 27 (36) | 2.8 (4.0) | 15 (26) | 9.1 (13) | [52] |
| Pontevedra (Spain) | ― | 4.4 (9.8) | 16 (28) | 6.0 (11) | 14 (27) | [53] |
| Tarcolas (Costa Rica) | ― | 6.5 (8.2) | ― | 2.8 (3.4) | 2.0 (2.3) | [54] |
| Sado (Portugal) | 4.4 (8.7) | 1.6 (32) | 10 (15) | 1.8 (2.7) | 5.4 (7.7) | [55] |
| Scheldt (The Netherlands) | 14 (63) | 2.3 (3.8) | 2.1 (4.7) | 3.5 (8.5) | 3.7 (7.7) | [56,57] |
| St Louis (Senegal) | 10 (16) | 2.2 (2.9) | 4.9 (8.1) | 27 (76) | ― | [58] |
| Susquehanna (United States) | 7.0 (9.2) | 1.0 (1.5) | 1.0 (2.5) | 5.0 (7.0) | 6.0 (8.6) | [59] |
| Sydney (Australia) | 11 (25)^b^ | 5.3 (14) | 8.3 (24) | 11 (20) | 8.5 (17) | This study |
|  | ― | 3.0 (10) | 14 (61) | 10 (57) | 11 (36) | [60,61] |
| Tamaki (New Zealand) | 3.6 (10) | ― | 5.2 (15) | 4.6 (7.8) | 3.7 (5.9) | [62] |
| Weser (Germany) | 10 (19) | ― | 2.1 (3.8) | 5.9 (8.5) | 2.9 (4.6) | [63] |
| Yangtze (China) | 3.3 (6.8) | 2.8 (5.6) | 2.4 (4.6) | 1.7 (2.3) | 3.6 (11) | [64,65] |
| **Mean** | **6.0** | **3.2** | **4.4** | **5.2** | **4.3** |  |

^a^ Mean (and maximum) values, based on iron- or aluminium-normalised sediment concentrations (µg/g dry weight).

^b^ Enrichment factors were calculated by dividing the aluminium-normalised metal sediment concentration in the Sydney Estuary by that from the Hawkesbury Estuary.

# References

[1] H. Windom, R. Smith, C. Rawlinson, M. Hungspreugs, S. Dharmvanu, G. Wattayakorn, Trace metal transport in a tropical estuary, Mar. Chem. 24 (1988) 293–305.

[2] D.J. Mackey, E.C. Butler, P.D. Carpenter, H.W. Higgins, J.E. O’Sullivan, R.B. Plaschke, Trace elements and organic matter in a pristine environment: Bathurst Harbour, Southwestern Tasmania, Sci. Total Environ. 191 (1996) 137–151.

[3] N.C. Munksgaard, D.L. Parry, Trace metals, arsenic and lead isotopes in dissolved and particulate phases of North Australian coastal and estuarine seawater, Mar. Chem. 75 (2001) 165–184.

[4] D. Tang, K.W. Warnken, P.H. Santschi, Distribution and partitioning of trace metals (Cd, Cu, Ni, Pb, Zn) in Galveston Bay waters, Mar. Chem. 78 (2002) 29–45.

[5] R.P. Trocine, J.H. Trefry, Metal concentrations in sediment, water and clams from the Indian river lagoon, Florida, Mar. Pollut. Bull. 32 (1996) 754-759.

[6] A.M. Cindrić, C. Garnier, B. Oursel, I. Pižeta, D. Omanović, Evidencing the natural and anthropogenic processes controlling trace metals dynamic in a highly stratified estuary: The Krka River estuary (Adriatic, Croatia), Mar. Pollut. Bull. 94 (2015) 199–216.

[7] R.T. Powell, W.M. Landing, J.E. Bauer, Colloidal trace metals, organic carbon and nitrogen in a southeastern U.S. estuary, Mar. Chem. 55 (1996) 165–176.

[8] M. Waeles, V. Tanguy, G. Lespes, R.D. Riso, Behaviour of colloidal trace metals (Cu, Pb and Cd) in estuarine waters: An approach using frontal ultrafiltration (UF) and stripping chronopotentiometric methods (SCP), Estuar. Coast. Shelf Sci. 80 (2008) 538–544.

[9] S.J. Markich, The Surface Water Chemistry of Coastal Rivers in South-eastern Australia, ASI C4/17. Aquatic Solutions International, Sydney (2017).

[10] R.E. Owens, P.W. Balls, Dissolved trace metals in the Tay Estuary, Estuar. Coast. Shelf Sci. 44 (1997) 421–434.

[11] W. Sirinawin, D.R. Turner, S. Westerlund, P. Kantharana, Trace metals study in the Outer Songkla Lake, Thale Sap Songkla, a southern Thai estuary, Mar. Chem. 62 (1998) 175–183.

[12] J. Fu, X.L. Tang, J. Zhang, W. Balzer, Estuarine modification of dissolved and particulate trace metals in major rivers of East-Hainan, China, Cont. Shelf Res. 57 (2013) 59–72.

[13] R.M. Gaschnig, R.L. Rudnick, W.F. McDonough, A.J. Kaufman, J.W. Valley, Z. Hu, S. Gao, M.L. Beck, Compositional evolution of the upper continental crust through time, as constrained by ancient glacial diamictite, Geochim. Cosmochim. Acta 186 (2016) 316–343.

[14] G. Birch, N. Shotter, P. Steetsel, The environmental status of the Hawkesbury River sediments, Aust. Geogr. Stud. 36 (1998) 37–57.

[15] M.A. Olmos, G.F. Birch, Application of sediment-bound metals in studies of estuarine health: a case study of Brisbane Water estuary, New South Wales, Aust. J. Earth Sci. 55 (2008) 641–654.

[16] C. Matthai, K. Guise, P. Coad, S. McCready, S. Taylor, Environmental status in the lower Hawkesbury-Nepean River, New South Wales, Aust. J. Earth Sci. 56 (2009) 225–243.

[17] J. Viers, B. Dupré, J. Gaillardet, Chemical composition of suspended sediments in World Rivers: New insights from a new database, Sci. Total Environ. 407 (2009) 853–868.

[18] O.A. Dahab, Chromium biogeochemical cycle in Abu Kir Bay, East of Alexandria, Egypt, Estuar. Coast. Shelf Sci. 29 (1989) 327–340.

[19] R.E. Cranston, J.W. Murray, The determination of chromium species in natural waters, Anal. Chim. Acta 99 (1978) 275–282.

[20] K.S. Abou-El-Sherbini, I.M. Kenaway, M.A. Hamed, R.M. Issa, R. Elmorsi, Separation and pre-concentration in a batch mode of Cd(II), Cr(III, VI), Cu(II), Mn(II, VII) and Pb(II) by solid-phase extraction by using silica modified with N-propylsalicylaldimine, Talanta 58 (2002) 289–300.

[21] G.E. Batley, J.P. Matousek, Determination of chromium speciation in natural waters by electrodeposition on graphite tubes for electrothermal atomization, Anal. Chem. 52 (1980) 1570–1574.

[22] T.L. Mullins, Selective separation and determination of dissolved chromium species in natural waters by atomic absorption spectrometry, Anal. Chim. Acta 165 (1984) 97–103.

[23] E.M. De Souza, A.L. Wagener, P. Farias, Voltammetric determination of Cr(III) and Cr(VI) in tropical estuarine waters: Advantages and limitations, Croat. Chem. Acta 70 (1997) 259–269.

[24] H. Badiei, J. McEnaney, V. Karanassios, Bringing part of the lab to the field: On-site chromium speciation in seawater by electrodeposition of Cr(III)/Cr(VI) on portable coiled-filament assemblies and measurement in the lab by electrothermal, near-torch vaporization sample introduction and inductively coupled plasma-atomic emission spectrometry, Spectrochim. Acta 78B (2012) 42–49.

[25] M.J. Gardner, J.E. Ravenscroft, Determination of chromium(III) and total chromium in marine waters, Fresenius J. Anal. Chem. 354 (1996) 602–605.

[26] E. Espada-Bellido, Z. Bi, C. van den Berg, Determination of chromium in estuarine waters by catalytic cathodic stripping voltammetry using a vibrating silver amalgam microwire electrode, Talanta 105 (2013) 287–291.

[27] K.E. Abu-Saba, A.R. Flegal, Chromium in San Francisco Bay: superposition of geochemical processes causes complex spatial distributions of redox species, Mar. Chem. 49 (1995) 189–199.

[28] J.L. Zhou, Y.P. Liu, P.W. Abrahams, Trace metal behavior in the Conwy estuary, North Wales, Chemosphere 51 (2003) 429–440.

[29] K.T. Jiann, L.S. Wen, P.H. Santschi, Three metal (Cd, Cu, Ni and Pb) partitioning, affinities and removal in the Danshei River estuary, macro-tidal, temporally anoxic estuary in Taiwan, Mar. Chem. 96 (2005) 293–313.

[30] G. Benoit, S.D. Oktay-Marshall, A. Cantu, E.M. Hood, C.H. Coleman, M.O. Corapcioglu, P.H. Santschi, Partitioning of Cu, Pb, Ag, Zn, Fe, Al, and Mn between filter-retained particles, colloids, and solution in six Texas estuaries, Mar. Chem. 45 (1994) 307–336.

[31] H. Takata, T. Aona, S. Uchida, Distributions of trace metals Co, Cu and Cd in northern Sagami Bay, Japan and their relationship to estuarine variables, Estuar. Coast. Shelf Sci. 111 (2012) 84–94.

[32] W. Sung, Some observations on surface partitioning of Cd, Cu, and Zn in estuaries, Environ. Sci. Technol. 29 (1995) 1302–1312.

[33] V. Hatje, G.F. Birch, D.M. Hill, Spatial and temporal variability of particulate trace metals in Port Jackson Estuary, Australia, Estuar. Coast. Shelf Sci. 53 (2001) 63–77.

[34] M.K. Koshikawa, T. Takamatsu, J. Takada, M. Zhu, B. Xu, Z. Chen, S. Murakami, K. Xu, M. Watanabe, Distributions of dissolved and particulate elements on the Yangtze estuary in 1997–2002: Background data before the closure of the Three Gorges Dam, Estuar. Coast. Shelf Sci. 71 (2007) 26–36.

[35] M. Caetano, C. Vale, M. Falcão, Particulate metal distribution in Guadiana estuary punctuated by flood episodes, Estuar. Coast. Shelf Sci. 70 (2006) 109–116.

[36] G.V. De Melo, J.A. Neto, O. Malm, M.A. Fernandez, S.M. Patchineelam, Composition and behavior of heavy metals in suspended sediments in a tropical estuarine system, Environ. Earth Sci. 73 (2015) 1331–1344.

[37] S. Suja, P.M. Kessarker, L.L. Fernandes, S. Kurian, A. Tomer, Spatial and temporal distribution of metals in suspended particulate matter of the Kali estuary, India, Estuar. Coast. Shelf Sci. 196 (2017) 10–21.

[38] R. Shynu, V.P. Rao, P.M. Kessarker, T.G. Rao, Temporal and spatial variability of trace metals in suspended matter of the Mandovi estuary, central west coast of India, Environ. Earth Sci. 65 (2012) 725–739.

[39] R. Prego, M.J. Belzunce, A. Cobelo, E. Helios-Rybicka, Particulate metal in the Ulla River estuary: State and sources of contamination (Arosa Ria, NW Iberian Peninsula), Cienc. Mar. 34 (2008) 381–388.

[40] B. Newman, S. Arabi, S. Weerts, R. Peters, N. Vogt, Prevalence and Significance of Organic Contaminants and Metals in Aquatic Ecosystems in the Ethekwini Area of Kwazulu-Natal, WRC Report 1977/1/15, Water Research Commission, Gezina (2015).

[41] G.O. Duodu, A. Goonetilleke, G.A. Ayoko, Potential bioavailability assessment, source apportionment and ecological risk of heavy metals in the sediment of Brisbane River estuary, Australia, Mar. Pollut. Bull. 117 (2017) 523–531.

[42] P.W. Balls, S. Hull, B.S. Miller, J.M. Pirie, W. Proctor, Trace metals in Scottish estuarine and coastal sediments, Mar. Pollut. Bull. 34 (1997) 42–50.

[43] D.G. Jones, C.H. Vane, S. Lass-Evans, S. Chenery, B. Lister, M. Cave, J. Gafeira, G. Jenkins, A. Leslie, N. Breward, K. Freeborough, I. Harrison, A.W. Kim, A. Lacinska, T. Milodowski, J. Ridgway, J. Riding, M. Strutt, D. Wagner, I. Wilkinson, Geochemistry and related studies of Clyde Estuary sediments, Earth Environ. Sci. Trans. R. Soc. Edinb. 108 (2019) 269–288.

[44] J.C. Fernandez-Cadena, S. Andrade, C.L. Silva-Coello, R. De la Iglesia, Heavy metal concentration in mangrove surface sediments from the north-west coast of South America, Mar. Pollut. Bull. 15 (2014) 221–226.

[45] C.M. Iwegbue, B. Lari, S.A. Osakwe, G.O. Tesi, G. Nwajei, S.S. Martincigh, Distribution, sources and ecological risks of metals in surficial sediments of the Forcados river and its estuary, Niger Delta, Nigeria, Environ. Earth Sci. 77 (2018) 227.

[46] E. Siddiqui, J. Pandey, Assessment of heavy metal pollution in water and surface sediment and evaluation of ecological risks associated with sediment contamination in the Ganga River: a basin-scale study, Environ. Sci. Pollut. Res. 26 (2019) 10926–10940.

[47] T. Nasrabadi, G.N. Bidhendi, A. Karbassi, N. Mehrdadi, Evaluating the efficiency of sediment metal pollution indices in interpreting the pollution of Haraz River sediments, southern Caspian Sea basin, Environ. Monit. Assess. 171 (2010) 395–410.

[48] A. Grant, R. Middleton, An assessment of metal contamination of sediments in the Humber Estuary, U.K, Estuar. Coast. Shelf Sci. 31 (1990) 71–85.

[49] M.S. Elias, S. Ibrahim, K. Samuding, S. Ab Rahman, Y.M. Wo, Assessment of toxic elements In sediments of Linggi River using NAA and ICP-MS techniques, MethodsX 5 (2018) 454–465.

[50] A. Coynel, L. Gorse, C. Curti, J. Schafer, C. Grosbois, G. Morelli, E. Ducassou, G. Blanc, G.M. Maillet, M. Mojtahid, Spatial distribution of trace elements in the surface sediments of a major European estuary (Loire Estuary, France): Source identification and evaluation of anthropogenic contribution, J. Sea Res. 118 (2016) 77–91.

[51] S.C. Decena, M.S. Arguelles, L.L. Robel, Assessing heavy metal contamination in surface sediments in an urban river in the Philippines, Pol. J. Environ. Stud. 27 (2018) 1–13.

[52] L.N. Castro, A.E. Rendina, M.J. Orgeira, Assessment of toxic metal contamination using a regional lithogenic geochemical background, Pampean area river basin, Argentina, Sci. Total Environ. 627 (2018) 125–133.

[53] R. Beiras, J. Bellas, N. Fernández, J.I. Lorenzo, A. Cobelo-Garcia, Assessment of coastal marine pollution in Galicia (NW Iberian Peninsula); metal concentrations in seawater, sediments and mussels (*Mytilus galloprovincialis*) versus embryo-larval bioassays using *Paracentrotus lividus* and *Ciona intestinalis*, Mar. Environ. Res. 56 (2003) 531–553.

[54] C.C. Fuller, J.A. Davis, D.J. Cain, P.J. Lamothe, T.L. Fries, G. Fernandez, J.A. Vargas, M.M. Murillo, Distribution and transport of sediment-bound metal contaminants in the Rio Grande de Tarcoles, Costa Rica (Central America), Wat. Res. 24 (1990) 805–812.

[55] M. Mil-Homens, C. Vale, J. Raimundo, P. Pereira, P. Brito, M. Caetano, Major factors influencing the elemental composition of surface estuarine sediments: The case of 15 estuaries in Portugal, Mar. Pollut. Bull. 84 (2014) 135–146.

[56] J.J. Zwolsman, G.T. van Eck, G. Burger, Spatial and temporal distribution of trace metals in sediments from the Scheldt Estuary, south-west Netherlands, Estuar. Coast. Shelf Sci. 43 (1996) 55–79.

[57] M. Bouezmarni, R. Wollast, Geochemical composition of sediments in the Scheldt Estuary with emphasis on trace metals. Hydrobiologia 540 (2005) 155–168.

[58] C. Diop, D. Dewaelé, F. Cazier, A. Diouf, B. Ouddane, Assessment of trace metals contamination level, bioavailability and toxicity in sediments from Dakar coast and Saint Louis estuary in Senegal, West Africa, Chemosphere 138 (2015) 980–987.

[59] S.A. Sinex, G.R. Helz, Regional geochemistry of trace elements in Chesapeake Bay sediments, Environ. Geol. 3 (1981) 315–323.

[60] S.E. Taylor, The Source and Remobilisation of Contaminated Sediment in Port Jackson, Australia, PhD Thesis. The University of Sydney, Sydney (2000).

[61] G.F. Birch, M.A. Olmos, Sediment-bound heavy metals as indicators of human influence and biological risk in coastal bodies, ICES J. Mar. Sci. 65 (2008)1407–1413.

[62] G.M. Abrahim, R.J. Parker, Assessment of heavy metal enrichment factors and the degree of contamination in marine sediments from Tamaki Estuary, Auckland, New Zealand, Environ. Monit. Assess. 136 (2008) 227–238.

[63] U. Förstner, W. Calmemo, J. Shoer, Heavy metals in bottom sediments and suspended material from the Elbe, Weser and Ems estuaries and from the German Bight (south eastern North Sea), Thalassia Jugosl. 18 (1982) 97–122.

[64] H. Feng, X. Han, W. Zhang, L. Yu, A preliminary study of heavy metal contamination in Yangtze River intertidal zone due to urbanization, Mar. Pollut. Bull. 49 (2004) 910–915.

[65] W. Zhang, H. Feng, J. Chang, J. Qu, H. Xie, L. Yu, Heavy metal contamination in surface sediments of Yangtze River intertidal zone: An assessment from different indexes, Environ. Pollut. 157 (2009) 1533–1543.
